# Graphene-Based Fiber Materials for Gas Sensing Applications: State of the Art Review

**DOI:** 10.3390/ma17235825

**Published:** 2024-11-27

**Authors:** Susanna Vu, Mohamed Siaj, Ricardo Izquierdo

**Affiliations:** 1 Department of Electrical Engineering, École de Technologie Supérieure, 1100 Rue Notre-Dame Ouest, Montréal, QC H3C 1K3, Canada; susanna.vu.1@ens.etsmtl.ca; 2 Department of Chemical Engineering and Biotechnological Engineering, Université de Sherbrooke, 2500 Boulevard de l’Université, Sherbrooke, QC J1K 2R1, Canada; mohamed.siaj@usherbrooke.ca

**Keywords:** gas sensor, fiber, nanofiber, fiber composite, graphene oxide, reduced graphene oxide, electrospinning, wet-spinning, gas sensing mechanism, flexible electronics

## Abstract

The importance of gas sensors is apparent as the detection of gases and pollutants is crucial for environmental monitoring and human safety. Gas sensing devices also hold the potential for medical applications as health monitoring and disease diagnostic tools. Gas sensors fabricated from graphene-based fibers present a promising advancement in the field of sensing technology due to their enhanced sensitivity and selectivity. The diverse chemical and mechanical properties of graphene-based fibers—such as high surface area, flexibility, and structural stability—establish them as ideal gas-sensing materials. Most significantly, graphene fibers can be readily tuned to detect a wide range of gases, making them highly versatile in gas-sensing technologies. This review focuses on graphene-based composite fibers for gas sensors, with an emphasis on the preparation processes used to achieve these fibers and the gas sensing mechanisms involved in their sensors. Graphene fiber gas sensors are presented based on the chemical composition of their target gases, with detailed discussions on their sensitivity and performance. This review reveals that graphene-based fibers can be prepared through various methods and can be effectively integrated into gas-sensing devices for a diverse range of applications. By presenting an overview of developments in this field over the past decade, this review highlights the potential of graphene-based fiber sensors and their prospective integration into future technologies.

## 1. Introduction

Gas sensors, devices designed to detect gases in a chosen environment, play a critical role in applications ranging from environmental monitoring to industrial systems monitoring and healthcare diagnostics [1,2,3,4,5]. Gas detection must be highly efficient, reliable, and accurate, especially when concerning the safety of both the individual and the environment [6]. For example, an occurrence such as a gas leak in an industrial setting can quickly become catastrophic and require immediate action [7], while the rapid detection and assessment of biomarkers must be precise for proper medical treatment [8]. Gas sensing of pollutants is also crucial in maintaining human health and safety, as well as ecosystem preservation and enabling effective regulation of harmful emissions [9,10,11,12]. The need for gas sensors in these settings has resulted in increasing sophistication in both gas sensor design and nanofabrication approaches [13]. In addition, as automated and remotely operated equipment becomes more widely adopted, gas sensors have been proposed to act as an electronic “nose” for these systems [14,15], underscoring the importance of the continued development of these sensors and their place in modern technologies [16].

Gas sensors can be categorized as physical and chemical sensors or have elements of both. Physical sensors rely on measurements of physical quantities, such as light or heat, while chemical sensors rely on measurement of chemical interactions at the gas–sensor interface [17]. Although the change in resistance is a physical measurement, the mechanism driving this effect may be physical absorption or chemical reaction, depending on the interactions between the gas and the sensing material [18,19]. Gas sensor performance can be influenced by the dimensionality of the sensor and the loading of the sensing materials [20,21]. A higher specific surface area of a sensing material leads to higher sensitivity, and several different fabrication techniques have been employed with the aim of producing sensors with intricate and fine structures to maximize surface area [22,23]. Many factors affect the gas sensing mechanism of a sensor, including the reactivity and atomicity of the gas, and the conductivity and morphology of the sensing material. Gas molecules can influence the response of a gas sensor due to the rate of diffusion of gas and the kinetics of collision between gas and surface [24,25]. Larger gas molecules may exhibit higher reactivity due to their greater collision diameters, enhancing interactions with the sensing material and generating a stronger response [26,27,28]. Conversely, smaller gas molecules can more easily penetrate the pores of the sensing material and diffuse more rapidly, resulting in faster response times [25,27,29]. These effects have led to efforts to tailor sensing materials by modifying surface properties [30,31,32] and tuning pore sizes to target specific gases based on molecular size [25,33,34,35]. As other areas of technology evolve, there is great interest in developing sensors with efficient sensing materials that are suited to these new technological landscapes [36,37]. A prominent example of this is the development of sensors that follow the design tenets of flexible and wearable electronics [38].

Challenges in developing flexible gas sensing platforms often involve selecting suitable sensing materials that can maintain their structure and functionality when strained [39]. Research focuses on optimizing these sensors to achieve comparable sensitivity to rigid substrates, along with high selectivity, fast response and recovery, and durability, ensuring consistent performance under repeated bending or stretching without damage [40]. Carbon-based materials offer mechanical stability and electronic properties and have been demonstrated to be promising sensitive materials for sensing applications [41]. Among these materials, graphene, defined as a single layer of sp^2^ carbon atoms, exhibits desirable characteristics and has been thoroughly investigated for use in sensors [42,43,44]. Many of the properties of graphene, including high conductivity, excellent chemical stability at ambient temperature, flexibility, intrinsic high surface area, and low fabrication costs make it an ideal candidate in gas sensing applications [45]. The large surface-to-volume ratio of graphene offers numerous active sites for gas adsorption, while its exceptional electrical conductivity enables fast response times, contributing to the sensitivity of the sensor. The sensitivity of graphene is such that it has been shown to be able to sense a single gas molecule [46].

Graphene materials, including pristine graphene, graphene oxide (GO), and reduced graphene oxide (RGO) [47], also demonstrate distinct gas detection capabilities that can be exploited in sensor fabrication [43]. GO can be produced at a low cost by chemically exfoliating graphite in the presence of a suitable oxidant [48]. GO has semiconducting properties superior to raw graphite and can be enhanced significantly by reduction to RGO using chemical, thermal, or UV reduction processes [43,48]. RGO is particularly advantageous as it possesses a defined band gap and has accessible functional groups capable of selectively binding gas molecules [48]. The implementation of graphene materials in composite fibers for gas sensing has garnered the most significant interest, as graphene and its derivatives have been proven to be robust nanofillers [49].

Graphene-based composite fibers can be fabricated with a wide range of materials including polymers, metal oxide nanoparticles, and even other 2D materials and synthetic fibers [17,50]. Combining graphene with other semiconducting materials, such as conductive polymers and metal oxides, often enhances sensing capabilities by increasing the surface available for gas interaction [3,51]. Graphene is a competitive gas sensing material due to its tolerance to humidity, unlike many polymers, and its broader operating temperature range, compared to some metal oxides [3]. Graphene-based fibers are advantageous functional materials as they benefit from the high sensitivity and stability of graphene, along with their good electrical and mechanical properties [3,43,52]. While harnessing the beneficial characteristics of graphene, graphene fibers provide increased flexibility and structural integrity, making them well-suited for sensor fabrication. With the growing interest in micro and nano gas sensors, graphene-based fibers are strong contenders owing to their conductivity, compatibility, conformability, and ease of integration into lightweight, flexible devices [53,54].

A popular method to fabricate nanofiber gas sensors is electrospinning, where an electrostatic force is employed to draw threads from a composite solution to form nanofibers [55]. Electrospinning offers an advantage over other nanofiber fabrication methods, such as chemical vapor deposition, sol-gel methods, and template-assisted fabrication, due to its simplicity, cost-effectiveness, and versatility relative to these competing nanofabrication techniques [17]. Another emerging technique for the fabrication of fibers is wet-spinning, whereby a composite material is extruded through a spinneret into a coagulation bath composed of a non-solvent [56,57]. In the coagulation bath, the material undergoes rapid drawing, resulting in fiber formation through phase inversion [57,58]. Electrospinning allows for more precise control of fiber diameter while wet-spinning can be more easily scalable [59,60]. Fibers produced through spinning methods are distinguished by their exceptionally high aspect ratio and surface area, as well as the ease with which graphene materials can be uniformly integrated throughout the fiber, enhancing the performance of the resulting sensor [61].

This review aims to provide an overview of the state-of-the-art graphene-based fiber materials for gas detection sensors, focusing on advancements from the past decade. This review will discuss both gas sensing systems designed to detect specific gases and those capable of detecting multiple gases. It will cover the synthesis and processing methods for these fiber sensors and delve into the sensing mechanisms, including response dynamics and modes of detection. To demonstrate the scope and selectivity of these gas sensors, the application of graphene-based fibers in sensing gases with varying atomicity (diatomic, triatomic, and polyatomic gases) is described to illustrate the various modes to target these different gases. Additionally, sensors designed to detect volatile organic compounds (VOCs), at times termed gas vapors, are discussed. In addition to sensors fabricated to sense one gas, detection systems for two or more gases are reviewed. These gases represent an extensive range of gases with respect to their molecular size, atomic composition, thermal conductivity, and oxidizing or reducing potential. This review distinguishes gas sensors designed for various target gases, to spotlight the range of sensing capabilities of graphene fibers and highlight their applicability in diverse settings and applications.

This review will focus on sensing systems that incorporate graphene and gas sensors with a graphene fiber component, examining how graphene was integrated and how it enhanced the overall performance of each system. This work will emphasize sensing systems that utilize graphene fibers; however, in some cases, gas sensing performance relies on the combination of graphene with other materials, such as metal oxides and polymers. In these instances, we will focus on the role of graphene, while briefly discussing its interaction with these materials in relation to sensor sensitivity. Throughout this review, research trends in this field will be revealed and breakthrough findings will be highlighted. By examining recent literature, it aims to showcase the potential of graphene-based composite fibers in gas sensing systems and illustrate how these advances set a foundation for their integration into diverse applications, including medical diagnosis, health management, environmental monitoring, and wearable electronics.

## 2. Graphene-Based Fiber Sensors for Diatomic Gases

It is imperative to detect diatomic gases, such as hydrogen (H_2_) and carbon monoxide (CO), for a plethora of reasons related to safety concerns that endanger human health and the environment. H_2_ is a non-toxic, colorless, odorless gas; however, it is primarily produced by fossil fuels and is highly flammable [17,62]. This is due to its low ignition energy, where even an H_2_ volume fraction of 4% in the air can trigger explosions [63,64]. H_2_ gas is also susceptible to leakage into the atmosphere, making its safe transportation and storage very challenging, especially in industrial settings [64]. Similarly to H_2_, CO is a colorless, odorless, and flammable gas generated from fossil fuels and industrial processes, as well as vehicle exhaust emissions and wildfires [17,65]. However, CO exposure is toxic to humans and can be lethal in high concentrations, while lower concentrations exposure can lead to adverse symptoms, including headache, nausea, and dizziness [65]. This occurs as CO binds to hemoglobin in blood with high affinity, competing with oxygen and reducing its capacity to carry oxygen by displacing it [66]. In addition to being colorless and odorless, both H_2_ and CO gas are less dense than air, which enables their accumulation in enclosed spaces; therefore, efficient sensors for these gases are essential [64,66].

### 2.1. H_2_ Gas Sensor

**Figure 1:**
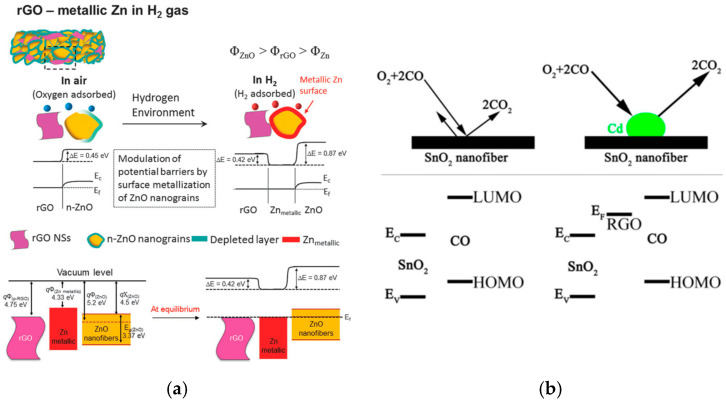
Examples of graphene-based fiber sensors for diatomic gases: (**a**) Schematic illustration of RGO-ZnO sensing mechanism for H_2_ gas [69]; (**b**) Schematic diagram of Cd/RGO/SnO_2_ sensing mechanism to CO gas [73].

While graphene alone has demonstrated effective gas-sensing properties, numerous studies are focused on optimizing these capabilities by combining graphene with other sensing materials [46,52,67]. Extensive research has focused on enhancing the sensitivity and selectivity of gas sensors by incorporating graphene and its derivatives with metal oxides, forming nanocomposite materials [50,68]. Kim et al. employed reduced graphene oxide to enhance the gas sensing capabilities of zinc oxide (ZnO) nanofibers for the selective detection of hydrogen gas [69]. The electrospun nanofibers were constructed from RGO-loaded ZnO, produced by incorporating RGO nanosheets with zinc acetate, and had an average diameter of 190 nm. The sensor exhibited the highest response of 2542 (*R_a_*/*R_g_*, where *R_a_* is the resistance in the absence and *R_g_* is the resistance in the presence of hydrogen) to 10 ppm H_2_ gas at 400 °C (Table 1). At the lowest concentration of H_2_ of 100 ppb, a response of 866 is shown by the sensor. The study suggests that there are electrical potential barriers at the interfaces of RGO/ZnO, RGO/Zn, and Zn/ZnO at equilibrium (Figure 1a). When introducing H_2_ gas to RGO-ZnO nanofibers, ZnO became n-type and was reduced to metallic Zn at the surface of the nanofiber, as hydrogen atoms reacted with surface oxygen ions of bulk ZnO. An energy potential barrier at the RGO/Zn interface prevented the flow of electrons into RGO, thus the addition and removal of H_2_ is a resistance modulation and generates a sensing signal. The high sensitivity of RGO-loaded ZnO nanofibers stands in contrast to SnO_2_ nanofibers fabricated in the same study, which are less responsive than the ZnO counterparts. Following similar methodologies, the same research group also fabricated RGO-ZnO nanofibers for sensing various other gases and reported their findings in a separate paper [70].

### 2.2. CO Gas Sensor

Incorporating graphene into a sensing system can amplify the overall electrical properties of the sensor, increasing its conductivity due to the high electron mobility of graphene [20,71]. Additionally, doping a graphene-based system with other semiconducting materials, or doping graphene itself, can further elevate the sensitivity and response time of the sensor, enabling more accurate and rapid detection of target gases [52,72]. Shams et al. electrospun cadmium-doped tin oxide and reduced graphene oxide composite nanofibers to function as a carbon monoxide gas sensor [73]. They reported the best-performing sensor, containing 1.6% cadmium, had a band gap of 2.80 eV and a diameter of 200.57 nm. In the presence of CO gas, the Cd-doped RGO-SnO_2_ nanofibers were able to respond after 25 s at 100 °C, while the nanofibers consisting of SnO_2_ alone showed a delayed response at 35 s (Table 1). The sensing mechanism of this system is enhanced by RGO due to its energy level, which lies between the LUMO orbital of CO and the conduction band of SnO_2_, and it exhibits p-type behavior relative to SnO_2_ (Figure 1b). This facilitates the transfer of electrons and decreases the resistance. In addition, the authors note that doping with Cd allowed for more sites for oxygen adsorption, which promoted the oxidation of Cd and generated more electrons back to the nanofiber. Therefore, a combination of RGO with Cd dopant further optimized the adsorption and desorption kinetics of the gas sensing system. To promote personal safety by minimizing potential hazards, this study presents a method for the rapid detection of toxic gases using sensors based on graphene-enhanced composites.

**Table 1:** Summary of graphene-based fiber gas sensors for H_2_ and CO gas.

Gas	Conc.	Material	Response	Temp.	Ref.
Hydrogen (H_2_)	100 ppm	RGO-ZnO	2542	400 °C	[69]
Carbon Monoxide (CO)	100 ppm	Cd/SnO_2_/RGO	25 s	100 °C	[73]

## 3. Graphene-Based Fiber Sensors for Triatomic Gases

Triatomic gases like carbon dioxide (CO_2_), hydrogen sulfide (H_2_S), and nitrogen dioxide (NO_2_) are gases that can come from anthropogenic sources, and high concentrations of each gas in the atmosphere are considered undesirable [74,75]. CO_2_ is a colourless and odourless gas and is the most prevalent greenhouse gas in our atmosphere [17]. The absorption of infrared radiation from the sun by atmospheric CO_2_ is understood to be the primary driver of climate change [76]. On a smaller scale, control of CO_2_ concentration in systems, enabled by quick and accurate measurements, is important in air quality, food preservation, and early fire detection [77]. Atmospheric CO_2_ levels remained at approximately 250 ppm from human evolution until the Industrial Revolution but doubled between the years 1813 and 2019 [78]. Prolonged exposure to CO_2_ levels up to 1000 ppm poses health risks to humans [79], making the detection and management of CO_2_ essential both indoors and outdoors. H_2_S is a highly toxic and flammable gas, largely generated from petroleum refineries and oil and gas drilling operations [17,80,81,82]. Exposure to H_2_S at low concentrations of 10 ppm should not exceed more than 10 min, while exposure to 100 ppm can cause instantons death [83]. Additionally, the distinct “rotten egg” odor of H_2_S is unpleasant; however, anosmia, or olfactory desensitization, can sometimes prevent the human olfactory system from detecting this gas. [84,85,86]. NO_2_ is a particularly important gas to detect as it is one of the primary emissions from the manufacturing and automobile industries [17]. Global average NO_2_ levels are on the rise, with motor vehicle exhaust contributing up to 80% of NO_2_ emissions in certain cities [87,88]. NO_2_ is associated with smog, and while not a greenhouse gas, it is toxic to humans, with the lethal dose being only 174 ppm [89]. NO_2_ gas detection is thus critical, especially in densely populated urban areas [77]. Given these factors, it is therefore essential to rapidly detect any present in the environment for the protection of individuals and the Earth. The presence of hazardous gases in the atmosphere, such as CO_2_, NO_2_, and H_2_S, necessitates constant environmental monitoring and emphasizes the need for high-performing gas sensors [90].

### 3.1. CO_2_ Gas Sensor

**Figure 2:**
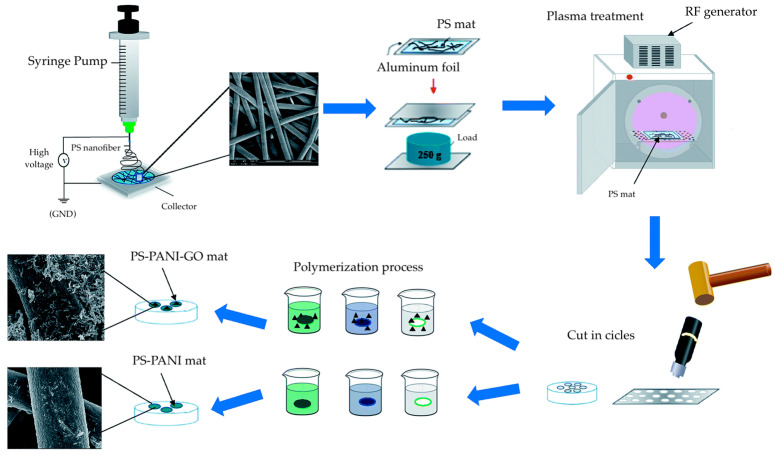
Schematic illustration of graphene/PANI/PS nanofibers preparation steps [91].

Al-Thani et al. reported CO_2_ sensors composed of polyaniline (PANI)-coated and RGO-PANI-coated electrospun polystyrene (PS) nanofibers, respectively [91]. The PS nanofibers underwent a plasma treatment, followed by coating with GO and PANI, whereby PANI was directly polymerized onto the fibers (Figure 2). The sensors containing GO were subjected to hydrogen reduction to convert to RGO with PANI-coated PS nanofibers. Although both sets of sensors showed sensitivity towards CO_2_ gas, the sensors containing RGO showed higher sensitivity, with a more distinct change in resistance, when exposed to 60 ppm of CO_2_ gas at room temperature. This behavior is credited to the broad electrochemical potential window and fast electron transfer rate of graphene [92]. The repeatability of the nanofiber sensors was examined, where the sensors exhibited a CO_2_ gas sensing response and recovery time of 65 s (Table 2). Furthermore, the selectivity of the nanofibers was investigated, where the RGO/PANI/PS sensor exhibited a high response of 0.8 (*(R_g_* − *R_a_*)/*R_a_*, where *R_g_* and *R_a_* are resistance in the presence of an analyte gas and N_2_, respectively) to CO_2_ and lower responses towards methanol, ethanol, and ammonia.

### 3.2. H_2_S Gas Sensor

**Figure 3:**
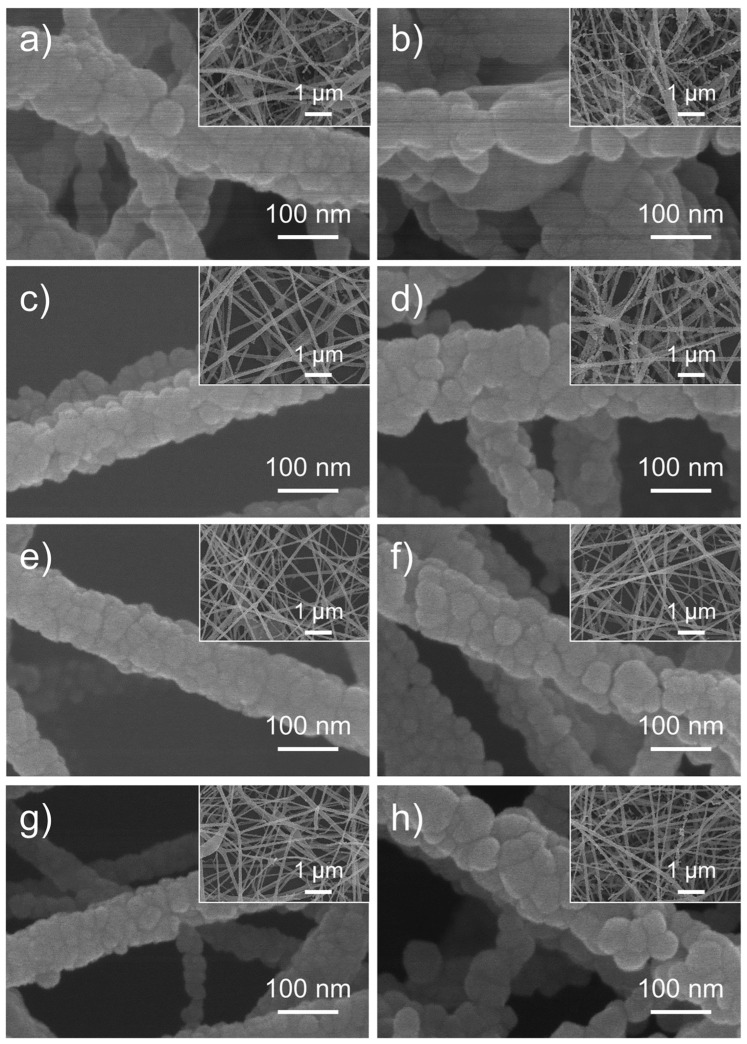
Field-emission scanning electron microscopy images of: (**a**) Pristine CuO nanofibers, and RGO-CuO nanofibers with different amounts of RGO; (**b**) 0.05 wt% RGO, (**c**) 0.1 wt% RGO, (**d**) 0.2 wt% RGO, (**e**) 0.3 wt% RGO, (**f**) 0.5 wt% RGO, (**g**) 1 wt% RGO, and (**h**) 1.5 wt RGO% [93].

Kim et al. fabricated H_2_S sensors using electrospun RGO-CuO nanofibers [93]. By testing RGO loadings ranging from 0.05 wt% to 1.5 wt%, they identified 0.5 wt% RGO-CuO as the optimal composition, which exhibited the highest sensitivity of 1.95 (*R_g_*/*R_a_*, where *R_a_* is resistance in air and *R_g_* is resistance in the presence of target gas) to 10 ppm of H_2_S at 300 °C. The RGO-CuO nanofibers also demonstrated selectivity for H_2_S when tested against other gases, including CO, C_6_H_6_, and C_7_H_8_, where the interfering gases showed minimal activity. The gas sensing efficiency was considerably influenced by the morphology of these nanofibers, which consist of nanoscale grains [94]. These nanograins play a critical role in determining the gas-sensing mechanism and enlarging the surface area of the fiber sensor. In the case of RGO-CuO, the varying RGO loadings alter the surface structure of the nanograins, leading to a distinct sensing response (Figure 3).

**Figure 4:**
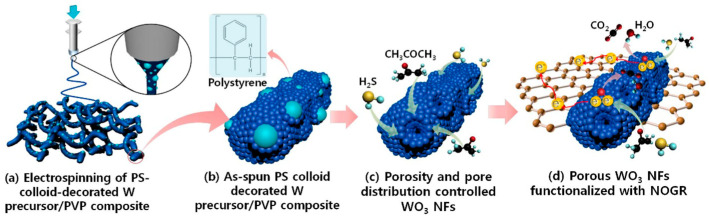
Schematic illustration of the fabrication process for PS-WO_3_/NOGR nanofibers, whereby controlled pore distribution on the nanofiber is achieved [95].

Kim et al. reported nanofibers for H_2_S detection based on non-oxidized graphene (NOGR), whereby pore size and distribution were controlled by a polymeric templating approach [95]. In the fabrication of their sensors, a composite containing colloidal polystyrene with tungsten precursor was electrospun, and the resulting nanofibers underwent a calcination procedure (Figure 4). In the process, the W precursor was oxidized, and PS colloids were decomposed. By tuning the size of the PS colloids, the size and distribution of pores can be controlled within the nanofibers as the PS colloids act as sacrificial templates and become void domains following the thermal treatment. In parallel, NOGR flakes were obtained through the chemical exfoliation of graphite intercalation compounds and subsequently combined with the PS-WO_3_ nanofibers. The resulting PS/WO_3_/NOGR nanofibers exhibited a sensitivity of 65.6 (*R_air_*/*R_gas_*) to 5 ppm of H_2_S gas at 300 °C (Table 2), and this was achieved with only 0.1 wt% loading of NOGR flakes. The selectivity of sensors was also examined by exposing the nanofibers to various gases, including acetone, NO, toluene, ethanol, NH_3_, CO, and pentane, where the sensor showed the highest response towards H_2_S. The conductivity of NOGR contributes to the sensing performance as it facilitates the transport of charge carriers, as well as the pores on the nanofibers that allow for higher surface area and gas penetration [96,97].

**Figure 5:**
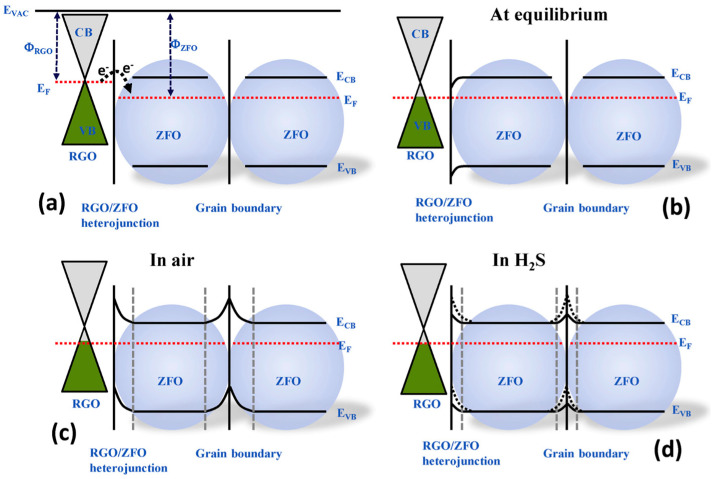
Schematic depicting the gas sensing mechanism of RGO-ZnFe_2_O_4_ nanofibers: (**a**) Band diagram of RGO and ZFO; (**b**) At equilibrium; (**c**) In air; (**d**) H_2_S gas exposure [98].

Hieu et al. developed RGO/ZnFe_2_O_4_ nanofiber sensors for H_2_S gas detection. The preparation of the sensors involved an on-chip electrospinning technique, wherein nanofibers were directly collected and assembled onto a microelectrode chip equipped with interdigitated electrodes [86]. The nanofiber sensors achieved a response of 147 (*R_a_*/*R_g_*, where *R_a_* and *R_g_* were the resistances of the sensors in the air and H_2_S, respectively) to 1 ppm of H_2_S at 350 °C (Table 2) [98]. The gas detection mechanism was attributed to the movement of electrons from RGO to ZnFe_2_O_4_ and the multi-porous structure of the sensor. The nanofibers were composed of nanograins, which induce the formation of depletion regions and potential barriers: one at the heterojunction between RGO and ZnFe_2_O_4_, and another at the boundaries between ZnFe_2_O_4_ nanograins (Figure 5). In the presence of air, oxygen molecules adsorb onto the surface of the nanofiber, capturing electrons from the conduction band to form oxygen ions. Upon exposure to H_2_S, the gas molecules react with these oxygen ions, generating the electrons back into the conduction band. This interaction reduces the heterojunction and grain boundary barriers, leading to a decrease in the resistance of the sensor. The response of the sensor occurs owing to the heterojunction between RGO and ZnFe_2_O_4_ within the nanofiber.

Hieu et al. extended their work on H_2_S gas sensors by fabricating RGO/α-Fe_2_O_3_ nanofiber sensors employing their previous on-chip electrospinning method [99]. The RGO/α-Fe_2_O_3_ nanocomposite was synthesized using poly(vinyl alcohol), a ferric salt precursor, and RGO reduced from GO. The nanofiber morphology was revealed to be significantly affected by changes in precursor concentration and annealing temperature while being independent of changes to the graphene content. The optimal sensor configuration, which yielded the highest response of 9.2 to 1 ppm H_2_S gas at 350 °C (Table 2), consisted of nanofibers containing 1.0 wt% RGO, 11 wt% PVA, and 4 wt% Fe(NO_3_)_3_·9H_2_O, and was annealed at 600 °C. The sensitivity of the sensor was attributed to the morphology of the RGO/α-Fe_2_O_3_ nanofibers and the presence of nanograins, along with the large surface-to-volume ratio provided by the RGO. It was noted that the sensing mechanism involved potential barriers at both heterojunctions and homojunctions, consistent with the mechanisms described in their previous work [98]. This study presents a straightforward approach for the detection of toxic gases and environmental monitoring.

Given rising concerns about air quality in our environment, accurate gas detection is critical in a multitude of settings. However, advancements must also focus on designing environmentally conscious sensing systems that can monitor gas in the environment without causing further harm to it. The environmental impact of graphene production itself must, therefore, also be evaluated. There are ongoing efforts to explore the production of graphite from biomass waste and the recycling of graphite from batteries to meet the demand for graphene [100,101]. With this, the lifespan of graphene-based sensors must be evaluated in future studies to ensure that they not only maintain optimal performance over their intended use but also that their materials can be reused to reduce the need for new production at the end of their operational life.

### 3.3. NO_2_ Gas Sensor

Promising improvements in sensor performance have been made by extending the investigation of polymeric substrates and polymer composites to electrospun nanofiber-based gas sensors [102,103]. Shi et al. reported reduced graphene oxide and polymer composite nanofibers for the fabrication of nitrogen dioxide gas sensors [104]. The electrospun nanofibers were composed of a poly(vinyl alcohol) (PVA) and poly(ether imide) (PEI) polymer mixture and deposited onto an interdigitated electrode. The nanofibers were then dip-coated in a GO nanosheet solution, enabling the self-assembly of GO onto the nanofibers and were subsequently reduced to form RGO-polymer nanofiber gas sensors. The sensor showed repeatability over multiple cycles with exposure to NO_2_ gas and N_2_, where it reached 90% of the maximum response (Δ*G*/*G*_0_, the ratio of conductance change of sensor in target gas to N_2_) upon exposure to 500 ppb of NO_2_. At the highest NO_2_ concentration of 5 ppm, the conductance increased by 159.4%, demonstrating that a higher NO_2_ concentration resulted in a greater sensing response. This trend was primarily attributed to the accessibility of the RGO surface to NO_2_ gas molecules [105,106].

Lee et al. also employed polymeric nanocomposites for the development of stretchable devices for the detection of NO_2_ gas [107]. They described sensors fabricated from RGO, where GO was chemically reduced with hydrazine, layered onto electrospun polyurethane (PU) nanofibers, and assembled on polydimethylsiloxane (PDMS). During the electrospinning process, the collected fibers were rotated to form nanofibers in orthogonal directions with varied electrospinning times and number of fiber layers. The mechanical stability of the sensors was tested by stretching at 50% elongation up to 10,000 cycles. When considering both stretchability and gas sensitivity, the overall best-performing sensor exhibited a response of 176% (Δ*I*/*I*, where *I* is the dynamic current intensity measured under stretching tests) to 5 ppm of NO_2_ gas at room temperature (Table 2) and was comprised of five layers that were electrospun for 8, 3, 3, 3, and 1 min. This study illustrated an approach toward high-performing wearable gas sensors that maintain sensing capabilities under high strains.

**Figure 6:**
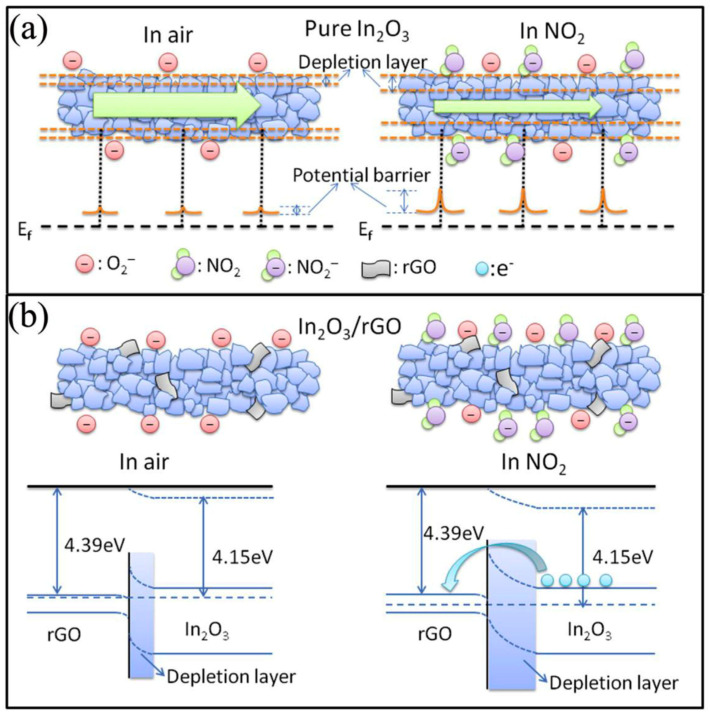
Schematic illustration of the sensing mechanism of (**a**) In_2_O_3_ compared to (**b**) RGO-In_2_O_3_ towards NO_2_ gas [111].

As previously mentioned, a widely popular approach to obtaining gas-sensing materials is through the formation of nanocomposites with graphene derivatives and metal oxides [108,109,110]. Wang et al. demonstrated this with the fabrication of RGO-In_2_O_3_ nanofiber gas sensors for NO_2_ detection [111]. These nanofibers were produced via electrospinning, incorporating In_2_O_3_ with RGO to form a composite material. The sensors containing 2.2 wt% RGO exhibited an enhanced gas response, with a sensitivity of 42 (*R_g_*/*R_a_*, where *R_g_* is the resistance of the sensor in NO_2_ and the *R_a_* is the sensor resistance in the air) to 5 ppm NO_2_ at 50 °C. The sensing mechanism of RGO-In_2_O_3_ nanofibers was influenced by the high surface area, structural defects, and functional groups of RGO, which provided ample adsorption sites for NO_2_ gas molecules [112,113]. Additionally, RGO enhances the resistance modulation of the sensor through the formation of RGO-In_2_O_3_ heterojunctions (Figure 6). When the sensor is in the open air, oxygen molecules are adsorbed at the surface and between the juncture of adjacent In_2_O_3_ nanoparticles at the nanograin boundaries. Potential barriers and depletion layers are formed at the nanograin boundaries between In_2_O_3_ nanoparticles, as well as between In_2_O_3_ and RGO heterojunctions when oxygen species are generated. When the nanofiber is exposed to NO_2_, the gas reacts with the adsorbed oxygen and expends electrons from the conduction band. This results in the expansion of the depletion layer, thereby altering the resistance of the sensor and producing a sensing signal.

The research group of Kim et al. are recognized for their work on RGO-loaded metal oxide electrospun nanofibers for gas sensing applications [69,70,93,114]. In two separate studies, the authors examined the gas sensing properties of electrospun RGO-SnO_2_ and RGO-ZnO nanofibers, evaluating their response to various oxidizing gases (NO_2_, SO_2_, O_2_) and reducing gases (CO, C_6_H_6_, C_2_H_5_OH) [70,114]. Although both nanofibers demonstrated sensitivity to a range of gases, the studies primarily focused on NO_2_ due to the notably high response observed. The high response was attributed to the inherent high reactivity of NO_2_ molecules as opposed to the selectivity of the nanofibers for NO_2_ [70,114].

**Figure 7:**
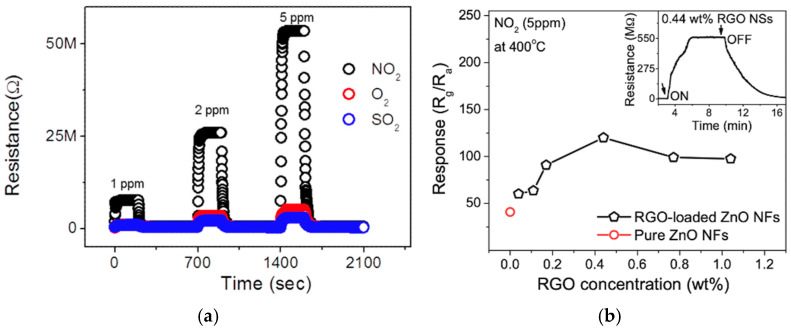
(**a**) Response of RGO-SnO_2_ nanofibers to NO_2_, O_2_, and SO_2_ gases, where the concentration was set to of 1, 2, and 5 ppm, respectively [114]; (**b**) Response of RGO-ZnO nanofibers, with varying RGO concentrations, to 5 ppm of NO_2_ gas [70].

In their investigations, nanofibers with RGO concentrations ranging from 0.04 to 1.04 wt% were evaluated. It was found that nanofibers with 0.44 wt% RGO exhibited the optimal sensing performance for both SnO_2_ and ZnO [70,114]. The authors suggest that increasing RGO concentrations beyond this optimal level reduced gas sensing performance due to percolation effects, wherein RGO forms conducting networks that interfere with the sensor. The optimal RGO concentration in the preparation of RGO-SnO_2_ nanofibers resulted in the most pronounced resistance modulation and a response of approximately 100 (*R_g_*/*R_a_*, where *R_g_* is the resistance of the sensor in NO_2_ and *R_a_* is the resistance in the air) when exposed to 5 ppm NO_2_ at 200 °C (Figure 7a, Table 2) [114]. Similarly, in the RGO-ZnO study, nanofibers with the same RGO loading exhibited the highest response, with a maximum response of 150 when exposed to 5 ppm NO_2_ at 400 °C (Figure 7b, Table 2) [70].

Scaling up the production of graphene-based fibers presents several challenges, particularly in transitioning spinning techniques to industrial-scale manufacturing. The studies discussed thus far all fabricated nanofibers using electrospinning methods, underlining the popularity of this technique for producing graphene fibers [49,115,116]. Despite the successful employment of this technique in the literature, it has been less widely adopted at an industrial scale due to its relatively slow production rates and challenges with maintaining consistency [117,118]. Wet-spinning fabrication techniques are currently regarded as a promising approach for the scalable production of microscale graphene composite fibers [57,119]. Although wet-spinning has been employed in the production of textiles such as viscose rayon fibers [120], adapting this technique for more complex functional materials remains an area of active research [60,121]. Further efforts are needed to optimize spinning techniques, enabling large-scale production while achieving precise control over fiber morphology and ensuring the functional performance of the fibers.

**Figure 8:**
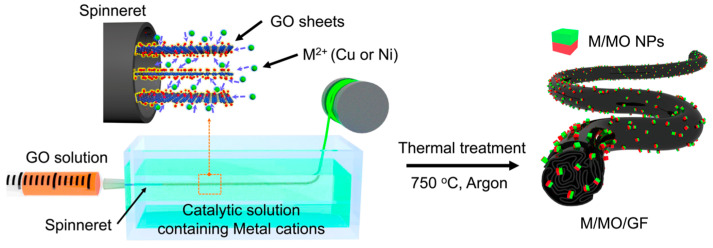
Schematic illustration of Cu/Cu_2_O/graphene and Ni/NiO/graphene fiber (M/MO/GF) fabrication, involving wet-spinning and thermal treatment of fibers [122].

Han et al. used a continuous wet-spinning technique to synthesize Cu-Cu_2_O and Ni-NiO graphene fibers [122]. In this process, a GO dispersion was extruded into a coagulation bath containing a catalytic solution with Cu or Ni ions, followed by thermal treatment of the fibers (Figure 8). The metal cations aid in binding the GO into fiber assemblies, making the wet-spinning technique suitable due to the even dispersion of cations in the coagulation bath. In addition, this preparation method allowed for sensors with flexibility and compatibility, enabling the fibers to be integrated into other fabrics. The resulting Cu/Cu_2_O/RGO and Ni/NiO/RGO fiber sensors demonstrated sensitivities of 18.90% and 0.82% (*(R_air_* − *R_gas_*/*R_air_*) × 100) respectively, to exposure of 5 ppm of NO_2_ gas at 150 °C (Table 2). Although the Ni/NiO/RGO fibers exhibited lower sensitivity compared to the Cu/Cu_2_O/RGO fibers, they outperformed other Ni-containing graphene fibers, showing double the response of NiO-graphene fibers. The gas sensing mechanism of the fibers involves the spillover effect, whereby adsorbed gas molecules are dissociated by metal into more reactive species and subsequently dispersed onto the adjacent surface [123,124].

**Figure 9:**
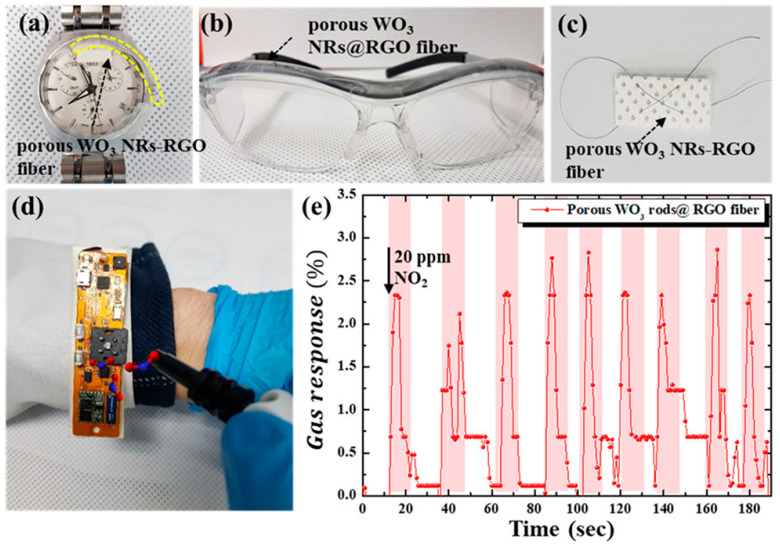
Images of RGO/WO_3_/TCNF fibers integrated into various objects: (**a**) A wristwatch (the area highlighted in yellow is where the fiber was integrated); (**b**) A pair of safety goggles; (**c**) Sown onto Kimtech paper; (**d**) A wearable sensing module. NO_2_ gas monitoring from the portable sensing device (**d**) is depicted in (**e**) [125].

Kim et al. also utilized the wet-spinning technique to fabricate graphene fibers, incorporating tunicate cellulose nanofiber (TCNF) with GO to create TCNF-GO fibers [125]. These wet-spun fibers were then treated with a tungsten (W) precursor and subjected to thermal processing, resulting in reduction and calcination to produce porous RGO/WO_3_/TCNF fibers. The inclusion of TCNF facilitated the formation of mesopores and created a wrinkled surface morphology, which increased the surface area of the fiber. The maximum response observed by the sensor was a sensitivity of 9.67% ((*R_air_* − *R_gas_*/*R_air_*) × 100) towards 5 ppm of NO_2_ at 100 °C (Table 2), although they remained functional at room temperature. To further demonstrate the practical application of these fibers, the authors integrated them into wearable devices, showcasing the potential of the fibers in wearable sensing systems (Figure 9).

**Figure 10:**
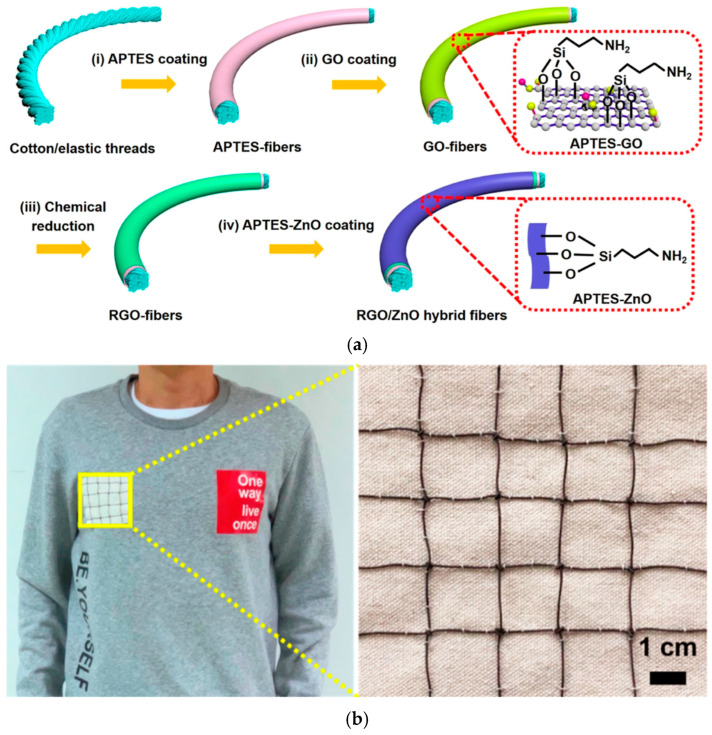
(**a**) Schematic illustration depicting the multi-step fabrication of RGO/ZnO/thread sensors; (**b**) Image of sensor array network of RGO/ZnO/thread and its integration onto wearable fabric [128].

Graphene can be utilized to elevate existing commercially available fiber materials, presenting straightforward approaches to high performance composites [126,127]. These graphene fiber composites not only offer cost-effective and widely accessible fabrication methodologies, but also have the potential to support large-scale production of wearable electronics [128,129]. Ren et al. developed RGO-enhanced mesoporous ZnO nanosheet hybrid fibers using cotton and elastic thread and evaluated their sensing response to NO_2_ gas [128]. The synthetic process involved treating the cotton and elastic treads with an adhesive and annealing treatment, followed by immersion in GO, a chemical reduction reaction, and subsequent coating with ZnO to produce RGO/ZnO/thread sensors (Figure 10a). These hybrid fibers demonstrated effective gas sensing capabilities, exhibiting a 44% response (*R* (*%*) = (*R_g_* − *R_a_*)/*R_a_* × 100, where *R_a_* is the initial resistance value in air and *R_g_* is the resistance value in NO_2_) to 15 ppm of NO_2_ at room temperature, with response and recovery times of 140 and 630 s, respectively (Table 2). To explore practical applications, the RGO/ZnO sensors were integrated into fabric, forming a wearable multi-sensor array network, which was successfully tested for NO_2_ detection (Figure 10b).

**Figure 11:**
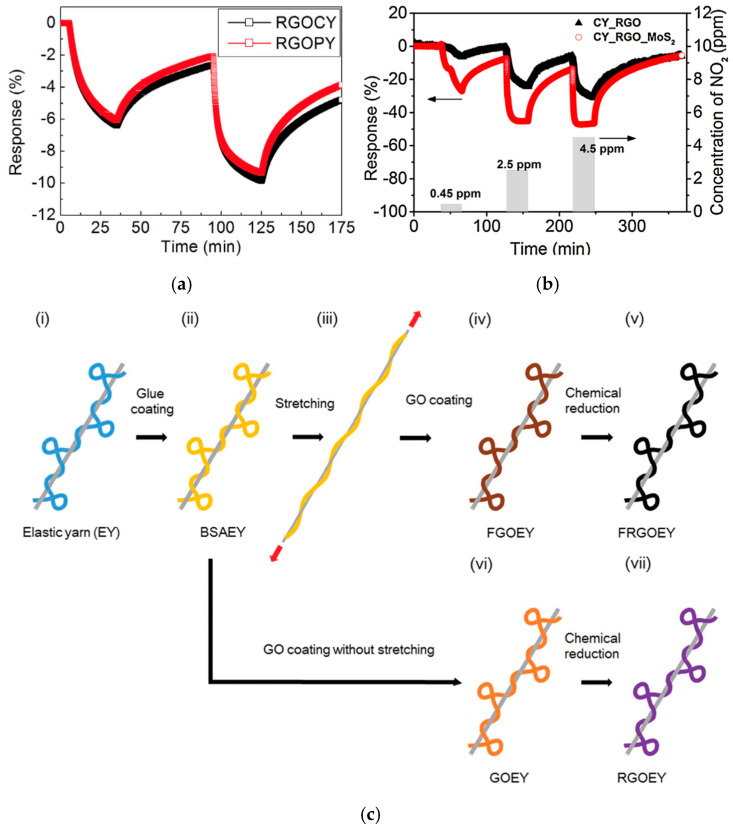

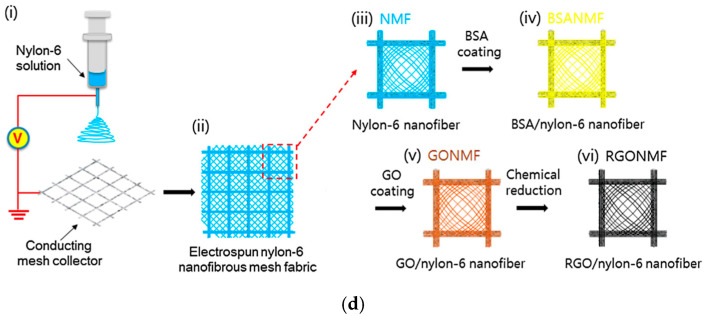
Examples of gas sensors using commercially available fibers: (**a**) Gas sensing performance of RGO-cotton yarn and RGO-polyester yarn exposed to 0.25 ppm and 1.25 ppm of NO_2_ at room temperature [129]; (**b**) Gas sensing performance of RGO-cotton yarn and RGO-cotton yarn with MoS_2_ exposed to 0.45 ppm, 2.5 ppm, and 4.5 ppm NO_2_ gas at room temperature [130]; (**c**) Schematic illustration of the fabrication process for RGO-elastic yarn [131]; (**d**) Schematic illustration of the fabrication process of RGO/nylon-6 [134].

The group of Lee et al. carried out extensive studies using commercially available fiber materials and coating them with graphene for various applications [129,130,131]. They reported the development of RGO-decorated cotton and polyester yarns [129]. The yarns were dip-coated in GO, which self-assembled onto the fibers. Subsequently, the GO-coated yarns were reduced to RGO through a low-temperature chemical reduction process. Utilizing these RGO fibers, they constructed devices capable of selectively detecting NO_2_ gas at concentrations as low as 0.25 ppm at room temperature. The RGO-cotton yarn sensors exhibited a response of −7.0% (*R* (%) = (*R_g_* − *R_a_*)/*R_a_* × 100, where *R_g_* and *R_a_* denote the electrical resistance upon exposure of NO_2_ and air, respectively), whereas RGO-polyester yarn sensors yielded a response −6.0% (Figure 11a, Table 2). When RGO-yarn sensors are exposed to NO_2_, the resistance of the sensors decreases. This decline was attributed to an increase in hole concentrations, resulting in the observed negative response.

Following their initial report, they investigated the use of cotton yarn coated with RGO and MoS_2_, utilizing similar processing methods [130]. This study revealed that incorporating MoS_2_ into RGO-containing fibers increased their sensitivity to NO_2_ by a factor of four compared to fibers containing only RGO. When exposed to 0.45 ppm of NO_2_, RGO/MoS_2_/yarn had a response of 28% (Δ*R*/*R*_0_ (%) = (*R_g_* − *R*_0_)/*R*_0_ × 100, where *R*_0_ and *R_g_* are resistances of the yarn sensor before and after exposure to NO_2_, respectively), while fibers without MoS_2_ only exhibited a response of 6% (Figure 11b, Table 2). This improved sensitivity was ascribed to the large surface area of the RGO-MoS_2_ composite and the synergistic interaction between RGO and MoS_2_ [132,133].

Building on their previous reporting, Lee et al. further examined the use of elastic yarn coated with RGO, employing techniques consistent with earlier studies (Figure 11c) [131]. In this investigation, the sensors demonstrated a response of 50–55% (*R* (%) = (*R_g_*− *R_a_*)/*R_a_* × 100, where *R_g_* and *R_a_* denote the electrical resistance upon exposure to NO_2_ and air, respectively) to 5 ppm of NO_2_ even under 200% strain (Table 2). Leveraging this performance, they fabricated wearable gas-sensing wristbands, thereby highlighting the potential of these RGO-coated fibers for integration into wearable electronics.

In another study led by Yun et al., nylon-6, a widely used industrial synthetic polymer, was fabricated into a mesh fabric through electrospinning [134]. This technique resulted in the fabrication of nanofibers, which were subsequently functionalized with GO using a self-assembly dip-coating method. Following this coating process, a chemical reduction was applied, converting the GO to RGO, thereby creating RGO/nylon-6 nanofibers (Figure 11d). The resulting nanofibers demonstrated sensitivity to NO_2_ gas, detecting concentrations at 1 ppm and exhibiting a response of 13.6% (|*R_g_* − *R*_0_|/*R*_0_, where *R*_0_ and *R_g_* are resistances of the gas sensor before and after exposure to NO_2_, respectively) at room temperature (Table 2). This response was attributed to the swelling of the hydrophilic and porous polymer, along with the high surface area of the nanofiber [135,136]. The bendability of the nanofibers was also examined, where a negligible change in response was observed for the sensors in flat and bent positions. These findings set a stage for the use of RGO-containing nanofibers in flexible electronics and electronic textiles applications.

**Table 2:** Summary of graphene-based fiber gas sensors for CO_2_, NO_2_, and H_2_S gas.

Gas	Conc.	Material	Response	Temp.	Ref.
Carbon dioxide (CO_2_)	60 ppm	RGO/PANI/PS	0.8	RT	[91]
Hydrogen Sulfide (H_2_S)	10 ppm	RGO-CuO	1.95	300 °C	[93]
	5 ppm	PS/WO_3_/NOGR	65.5	300 °C	[95]
	1 ppm	RGO-ZnFe_2_O_4_	147	350 °C	[98]
	1 ppm	RGO/α-Fe_2_O_3_	9.2	350 °C	[99]
Nitrogen Dioxide (NO_2_)	5 ppm	RGO/PVA/PEI	159.4%	RT	[104]
	5 ppm	RGO-PU	176%	RT	[107]
	5 ppm	Cu/Cu_2_O/RGO	18.90%	150 °C	[122]
	5 ppm	Ni/NiO/RGO	0.82%	150 °C	[122]
	5 ppm	RGO/WO_3_/TCNF	9.67%	100 °C	[125]
	5 ppm	RGO-In_2_O_3_	42	50 °C	[111]
	5 ppm	RGO-SnO_2_	100	200 °C	[114]
	5 ppm	RGO-ZnO	150	400 °C	[70]
	15 ppm	RGO/ZnO/thread	44%	RT	[128]
	0.25 ppm	RGO-cotton yarn	−7.0%	RT	[129]
	0.25 ppm	RGO-polyester yarn	−6.0%	RT	[129]
	0.45 ppm	RGO/MoS_2_/yarn	28%	RT	[130]
	5 ppm	RGO-elastic yarn	55%	RT	[131]
	1 ppm	RGO/nylon-6	13.6%	RT	[134]

## 4. Graphene-Based Fiber Sensors for Polyatomic Gases

The polyatomic gas, ammonia (NH_3_), is an important feedstock for fertilizer, energy, and fine chemicals [137]. NH_3_ is toxic and exposure to the gas should be restricted to 35 ppm for 10 min [138]. Other polyatomic gases, such as methane (CH_4_) and propyl radical (C_3_H_7_), which are components of natural gas, have thermal conductivities that differ significantly from that of air, making calorimetric gas sensors highly effective for detecting these gases [139,140]. However, the thermal conductivity of NH_3_ is similar to that of air, and detection of this gas by measuring thermal conductivity is difficult [139]. Substantial efforts have therefore been dedicated to the gas sensing of NH_3_ gas using alternative methods [139,141]. In addition, as the normal concentration of NH_3_ in a healthy person ranges from 0.5 to 2 ppm, NH_3_ in human breath has been explored as a biomarker, showing considerable potential for liver and kidney disease screening [141,142].

### NH_3_ Gas Sensor

**Figure 12:**
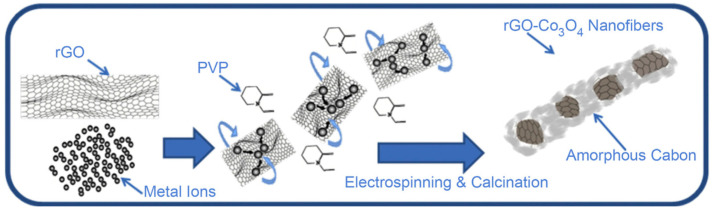
Schematic illustration of the fabrication process for RGO–Co_3_O_4_ nanofibers involving electrospinning and calcination process [143].

Gaskov et al. developed NH_3_ gas sensors by encapsulating Co_3_O_4_ nanocrystals within a matrix of RGO [143]. The nanofibers were synthesized by combining cobalt, GO, and polyvinyl pyrrolidone (PVP). During the electrospinning process, GO enveloped cobalt ions, while PVP formed the nanofiber structure (Figure 12). Subsequent calcination resulted in the reduction of GO to RGO, carbonization of PVP into amorphous carbon, and the aggregation of cobalt oxide into larger crystals, ultimately forming RGO-Co_3_O_4_ nanofibers. The sensor demonstrated a sensitivity of 53.6% (*R_g_* − *R_a_*)/*R_g_*) to 50 ppm of NH_3_ at room temperature (Table 3). The strong affinity of RGO for NH_3_ contributed to the response of the sensor [144,145]. When NH_3_ is adsorbed onto the nanofiber surface, it donates electron density in the relatively high energy lone pair orbital to the sp^2^ carbon of graphene, which increases resistance by reducing the number of electron holes.

Expanding on this work, Wang et al. reported nanofibers of amorphous carbon and Co_3_O_4_ encapsulated graphene for NH_3_ sensing [146]. In their study, a mixture containing GO, Co_3_O_4_, PVP, and a cobalt salt precursor was electrospun to form nanofibers. These nanofibers were then calcined, followed by an additional thermal treatment. The nanofibers subsequently underwent a carbon exfoliation process for varying durations, ranging from 0 to 580 s, during which amorphous carbon and RGO aggregated around the cobalt ions, forming carbon/RGO/Co_3_O_4_ nanofibers. It was observed that the sensor thermally etched for 250 s exhibited the highest response, with a 123% (*R_g_* − *R_a_*)/*R_a_*) sensitivity to 50 ppm of NH_3_ at room temperature (Table 3). Compared to the earlier work by Gaskov et al., the carbon/RGO/Co_3_O_4_ sensors demonstrated a significant improvement in performance, with over a 50% increase in sensitivity achieved through optimized carbon exfoliation.

**Figure 13:**
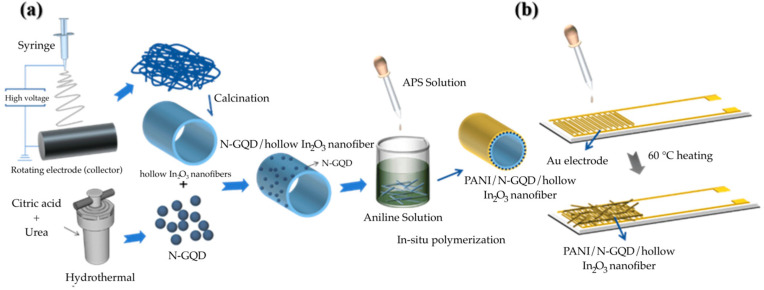
Schematic illustration of (**a**) The preparation of hollow In_2_O_3_ nanofibers, N-GQDs, and PANI/N-GQD/In_2_O_3_ nanofibers, and (**b**) nanofiber sensor fabrication [147].

Wu et al. developed nanofiber sensors for NH_3_ gas based on polyaniline, nitrogen-doped graphene quantum dots (N-GQD), and In_2_O_3_ [147]. The N-GQDs were synthesized through a hydrothermal process, while hollow In_2_O_3_ nanofibers were fabricated via electrospinning (Figure 13). The N-GQDs were subsequently combined with In_2_O_3_ nanofibers through electrostatic interaction, and PANI/N-GQD/In_2_O_3_ nanofibers were prepared through in-situ chemical oxidative polymerization. The assembly of the nanofiber sensors was completed by depositing them onto gold-interdigitated electrodes. In assessing the effect of N-GQD loading, it was found that sensors with 20 wt% N-GQD exhibited the highest response, achieving a sensitivity of 15.2 (*R_g_*/*R_a_*) to 1 ppm NH_3_ at room temperature (Table 3). This enhanced sensitivity was attributed to the increased surface area provided by the N-GQDs and hollow In_2_O_3_ nanofibers, which facilitate greater interaction with PANI and offer numerous adsorption sites for NH_3_ gas. Notably, the sensor demonstrated effective NH_3_ gas detection at room temperature concentrations ranging from 0.6 ppm to 2.0 ppm, the range in which kidney or liver diseases can be identified in human breath [148,149].

**Figure 14:**
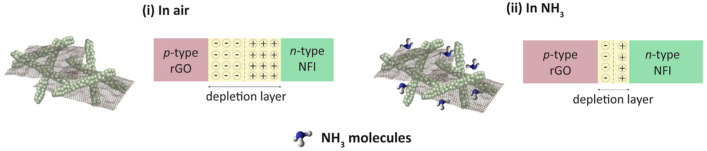
Schematic representation of RGO-In_2_O_3_ gas sensing mechanism, depicting depletion layer in air (**i**) and in NH_3_ gas (**ii**) [150].

Correa et al. also employed In_2_O_3_ in their preparation of sensors for NH_3_ detection [150]. In their study, In_2_O_3_ nanofibers were fabricated via electrospinning, followed by a calcination procedure, while RGO was synthesized by partial chemical reduction of GO using sodium citrate. RGO was combined with In_2_O_3_ via ultrasonication to obtain RGO-In_2_O_3_ nanofibers, which were then cast onto gold interdigitated electrodes to form gas sensing devices. The sensors displayed a sensitivity of 95% ([(*R_a_* − *R_g_*)/*R_g_*] × 100, where *R_a_* is the sensor resistance in air while *R_g_* is the sensor resistance after being exposed to the gas) in response to 15 ppm of NH_3_ gas at room temperature (Table 3). The sensors demonstrated selectivity for NH_3_, showing a higher response to it compared to other gases such as acetone, ethanol, methanol, triethylamine, trimethylamine, and monomethylamine. The gas sensing performance is attributed to the formation of a depletion layer and p-n heterojunction between RGO and In_2_O_3_, where oxygen molecules from the air are adsorbed onto the surface of the nanofiber and electronics flow from n-type In_2_O_3_ to p-type RGO until equilibrium is reached (Figure 14). When exposed to NH_3_, gas molecules react with the oxygen species, which generate electrons and eject them back into the nanofiber, thereby decreasing the resistance of the sensor and providing a sensing signal. RGO contributes to this response as it inherently provides the sensor with active sites at the surface and allows for more gas adsorption as it creates an interconnected structure with In_2_O_3_ [151,152].

**Figure 15:**
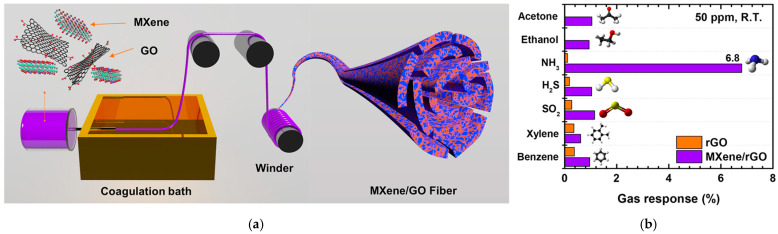
(**a**) Schematic illustration of MXene/GO via wet-spinning; (**b**) Selectivity of RGO/Ti_3_C_2_T_x_ MXene to NH_3_ in comparison to other gases [153].

Han et al. also contributed advancements in NH_3_ gas sensors through their work on wet-spun RGO/Ti_3_C_2_T_x_ MXene hybrid fibers [153]. In addition to previously mentioned metal oxides, Ti_3_C_2_T_x_ MXene, an emerging class of 2D material, is also recognized for its facilitation of gas adsorption [154,155]. The Ti_3_C_2_T_x_ was synthesized by etching Ti_3_AlC_2_ and combined with GO, then the resulting composite was wet-spun into fibers and subjected to thermal reduction (Figure 15a). MXene and GO undergo galvanic displacement, where oxygen atoms from GO transfer to the MXene surface, while electrons of MXene reduce GO, driven by the difference in their relative potentials [156,157]. The sensors demonstrated a sensitivity of 6.77% (Δ*R*/*R*_0_) in response to 50 ppm of NH_3_ at room temperature (Table 3). When tested for selectivity, the fibers demonstrated a notably higher response to NH_3_, while sensitivity to other gases remained low at approximately 1%. (Figure 15b). The potential of these fibers as wearable sensors was further explored by integrating them into a lab coat. The woven RGO/Ti_3_C_2_T_x_ MXene sensor demonstrated a response of 7.21% when exposed to 100 ppm of NH_3_ gas, underscoring the promise of these fibers for use in wearable and flexible sensing devices.

**Figure 16:**
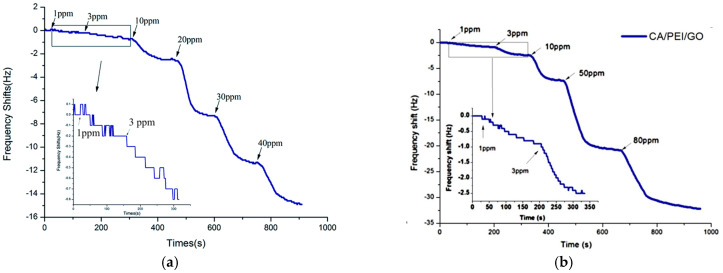
(**a**) Frequency shifts of G-COOH/PS QCM sensors upon exposure to increasing NH_3_ concentrations [158]; (**b**) Frequency shifts of CA/PEI/GO QCM sensors upon exposure to increasing NH_3_ concentrations [162].

Dong et al. introduced an innovative approach to fabricating coatings for a quartz crystal microbalance (QCM), a technique used to determine the mass of an analyte absorbed by measuring the frequency changes related to adsorption activity on a quartz crystal [158,159]. They developed an NH_3_ gas sensor by utilizing electrospun nanofibers made from polystyrene doped with carboxyl graphene (G-COOH) as the QCM coating [158]. G-COOH was specifically chosen for its high surface area and porosity, which serve to enhance the mass loading of NH_3_ molecules onto the QCM [160,161]. The G-COOH and PS composite was electrospun and directly deposited onto the QCM, forming a G-COOH/PS/QCM sensor. When tested with NH_3_ concentrations ranging from 1 to 40 ppm at room temperature, the sensor exhibited a decrease in frequency as ammonia concentration increased (Figure 16a). The sensor, exhibiting an inherent frequency of 5 MHz, achieved a sensitivity of 17.67 ng Hz^−1^.

This work was further expanded upon by Li et al., as they proposed that improved dispersibility of graphene could enhance the response of the sensor [162]. They described their approach as an electrostatic layer-by-layer self-assembly technique, whereby negatively-charged electrospun cellulose acetate (CA) nanofibers were encased with a layer of positively-charged poly(ether imide) and a layer of negatively-charged GO. The resulting nanofiber membrane was utilized as sensing coatings for QCM to form CA/PEI/GO/QCM NH_3_ gas sensors. With this modified method, the inherent frequency of the sensor was 5 MHz and the sensitivity of the sensor increased to 53.01 ng Hz^−1^ (Figure 16b). When exposed to 1 ppm of NH_3_, the sensor observed a higher response of 0.9 Hz, compared to a response of 0.3 Hz by the previously reported G-COOH/PS QCM sensor (Table 3) [158]. This effect was attributed to the improved uniformity of GO, which was more evenly distributed on the nanofiber as a coating, rather than being mixed into a spinning solution. The authors also attributed this enhanced sensitivity to the 3D structure of the layered fiber, with the CA nanofibers providing permeable space for gaseous NH_3_ molecules.

**Table 3:** Summary of graphene-based fiber gas sensors for NH_3_ gas.

Gas	Conc.	Material	Response	Temp.	Ref.
Ammonia (NH_3_)	50 ppm	RGO-Co_3_O_4_	53.6%	RT	[143]
	50 ppm	Carbon/RGO/Co_3_O_4_	123%	RT	[146]
	1 ppm	PANI/N-GQD/In_2_O_3_	15.2	RT	[147]
	15 ppm	RGO-In_2_O_3_	95%	RT	[150]
	50 ppm	Ti_3_C_2_T_x_ MXene/RGO	6.77%	RT	[153]
	1 ppm	G-COOH/PS/QCM	0.3 Hz	RT	[158]
	1 ppm	CA/PEI/GO/QCM	0.9 Hz	RT	[162]

## 5. Graphene-Based Fiber Sensors for Volatile Organic Compounds

Volatile organic compounds (VOCs) are a class of organic compounds that vaporize and aerosolize readily due to a relatively high vapor pressure at standard temperature and pressure [163]. VOCs are emitted from both natural and anthropogenic sources, with several being acutely toxic to humans [164]. Their release into the atmosphere can also lead to the formation of harmful secondary pollutants [165], thus there is a need to monitor levels of these compounds to assess indoor and outdoor air quality. Formaldehyde is a toxic VOC found in common products like paint and preservatives, while chlorobenzene, although less prevalent, is associated with carcinogenic effects [166,167]. Additionally, the emerging interest in using certain gases as biomarkers for human health highlights the importance of detecting and quantifying these gases [168,169,170,171]. Analyzing breath samples for the presence or absence of VOCs, such as acetone and ethanol, has shown that they are useful indicators of disease and adverse health conditions [172].

### 5.1. Acetone

Wang et al. realized porous GO-WO_3_ electrospun nanofibers for the gas sensing of acetone [173]. In this study, various volumes of GO ranging from 0 to 1.5 mL were added to a tungsten precursor solution and processed using electrospinning. The resulting nanofibers were calcined to obtain CO-WO_3_ nanofiber sensors. The sensor exhibiting the highest response contained nanofibers fabricated from 1 mL of GO-WO_3_, with a sensitivity of 35.9 (*R_a_*/*R_g_*) to 100 ppm of acetone vapor at 375 °C (Table 4). The enhanced sensing performance of GO-WO_3_ nanofibers is primarily due to the formation of ohmic contact between conductive GO nanosheets and WO_3_ nanograins, which facilitates electron migration and resistance modulation [174]. The morphology of the nanofibers, including high surface area and porosity, enables better adsorption and faster diffusion of acetone molecules, leading to quicker response and recovery times [175].

**Figure 17:**
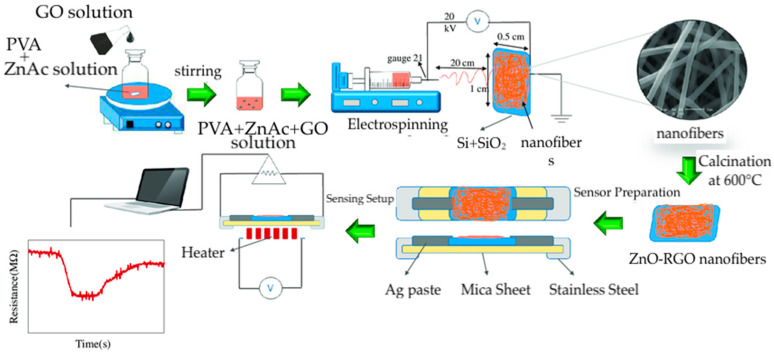
Schematic illustration of the preparation of RGO-ZnO nanofiber sensors [176].

Ghafarinia et al. developed RGO-ZnO nanofibers for the detection of acetone gas via electrospinning [176]. The nanofibers were prepared with different ratios of zinc acetate and GO, facilitated by PVA, followed by a calcination treatment (Figure 17). The sensors were fabricated by depositing the nanofibers onto a silicon wafer. It was determined that the sensor containing a zinc acetate concentration of 4 weight fractions and a GO concentration of 0.07 weight fractions performed the best. The sensor exhibited a sensitivity of 4 (*R_air_*/*R_gas_*) to 200 ppm of acetone at 200 °C (Table 4). Interestingly, a study of the sensors containing ZnO without RGO revealed that the addition of graphene decreased the optimal operating temperature from 400 °C to 200 °C. This improvement was attributed to the efficient charge transfer capabilities of RGO that refine electrical conductivity [177].

Lu et al. reported RGO/α-Fe_2_O_3_ nanofibers for acetone gas detection [178]. The nanofibers were fabricated with different loadings of RGO via electrospinning. The optimal sensor, containing 1 wt% of RGO/α-Fe_2_O_3_ nanofibers, reached a maximum of 8.9 (*R_a_*/*R_g_*) to 100 ppm acetone at 375 °C (Table 4), which was 4.5 times higher than the sensors without RGO. The formation of RGO and Fe_2_O_3_ heterojunctions generated ohmic contacts, enhancing the sensing signal, while defects and functional groups in RGO provided strong adsorption sites for gas molecules. The sensing mechanism is also influenced by the catalytic effect of RGO in adsorption, where the pores between layers of RGO nanosheets are efficient gas diffusion channels, offering active sites for acetone gas molecules [51].

**Figure 18:**
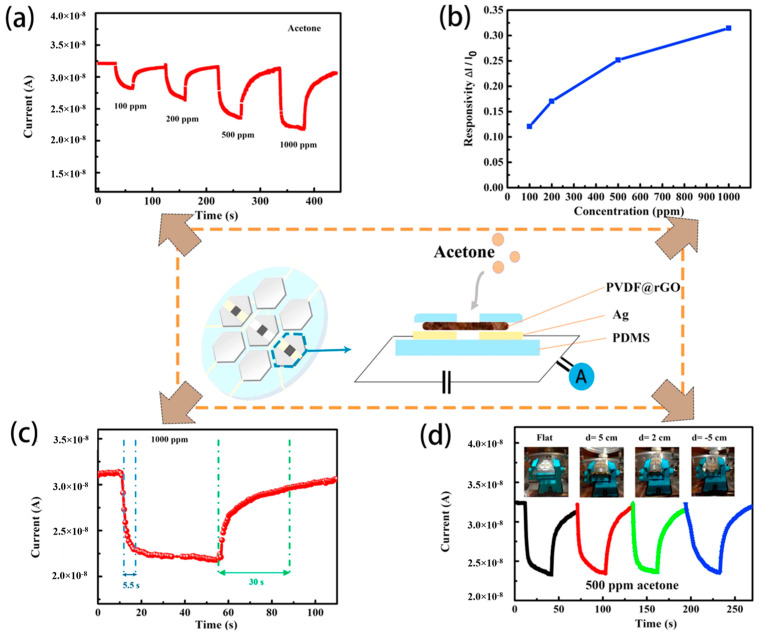
Gas sensor on e-skin device: (**a**) Response of gas sensor to different concentrations of acetone vapor; (**b**) Response of gas sensor to increasing acetone concentrations; (**c**) Response and recovery time of gas sensor; (**d**) sensing stability of device under different bending states in 500 ppm of acetone vapor (each color on the plot represents the response for different bending states) [179].

Shen et al. recognized the potential of RGO-poly(vinylidene fluoride) (PVDF) nanofibers for sensing and energy storage applications [179]. The nanofibers were fabricated by electrospinning a composite of PVDF and GO, followed by the reduction of GO to RGO using hydrazine, resulting in RGO-PVDF nanofibers. These nanofibers were employed in the fabrication of three sensor types, including pressure, photodetector, and gas sensors, as well as three micro-supercapacitors. For each sensor, nickel film electrodes were placed on two ends of the nanofibers at different distances between the electrodes, depending on the type of sensor. All device types were integrated onto a single PDMS substrate with thermally evaporated Ni and Ag tape electrodes serving as electrical interconnections. The entire structure was then encapsulated with an additional PDMS layer, exposing only the sensing materials to air, to create a self-powered multifunctional electronic skin system. The gas sensing function of the electronic skin (e-skin) demonstrated a response of 0.25 (*S* = Δ*I*/*I*_0_, where Δ*I* is the difference current between in the air and in the target gas, *I*_0_ is the current in the air) to 500 ppm of acetone at room temperature (Table 4), with rapid response and recovery times of 5.5 and 30 s, respectively (Figure 18). This work establishes a promising platform for advancing e-skin technologies and integrating sensors into wearable devices.

### 5.2. Chlorobenzene

**Figure 19:**
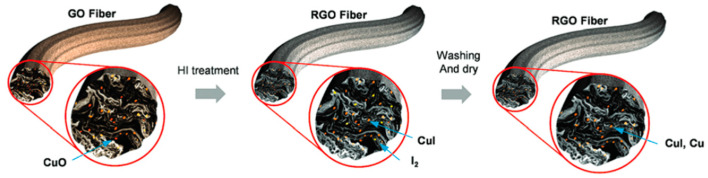
Schematic depicting RGO-Cu fiber preparation illustrating the content of Cu in the fiber [180].

Park et al. described chlorobenzene gas sensors made from RGO fibers embedded with copper iodide and metallic copper, developed using an innovative wet-spinning method [180]. They began with a GO liquid crystal dispersion, extruding it into a CuCl_2_-ethylene glycol coagulation solution where the GO aligned due to shear forces and formed fibers with Cu cations through ionic and van der Waals interactions (Figure 19). A portion of the Cu cations is converted to copper hydroxide, which was subsequently reduced to metallic Cu via a redox reaction with hydrogen iodide and acetic acid. This process led to the formation of CuI alloys, with any residual iodine rinsed away. The resulting RGO-Cu fibers served dual purposes as both gas and temperature sensors, where the response of the sensors was measured by the change in conductance (ΔG) upon varying rates of chlorobenzene evaporation. As gas sensors, the fibers showed increased conductance in response to rising chlorobenzene evaporation rates, with sensors containing higher Cu concentrations displaying greater conductance change at 20 °C. The conductance of the sensor reached 12.5 × 10^−6^ G when exposed to chlorobenzene vapor, with response and recovery times of approximately 70 s (Table 4). The gas sensitivity of RGO-Cu fibers is likely due to the activation of surface oxygen ions in the encapsulated oxidized Cu particles. The change in conductance results from gas molecules absorbed on the surface of Cu, which increase hole conductivity by accepting electrons from the oxygen ions. Additionally, these electrical properties and large accessible surface area for gas activity are promoted by RGO in the fiber sensor [181].

### 5.3. Ethanol

**Figure 20:**
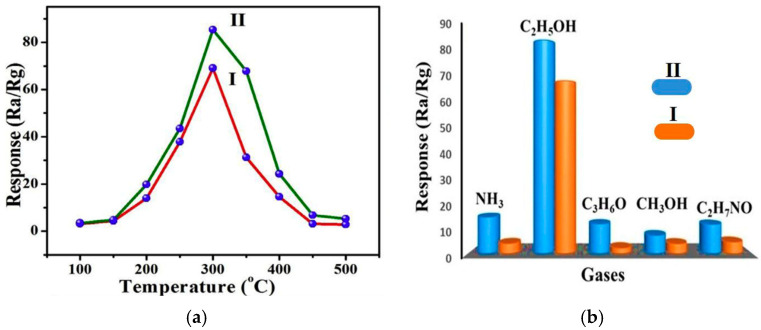
(**a**) Response of GO-SnO_2_ nanofibers (II) and SnO_2_ without GO (I); (**b**) Selectivity of GO-SnO_2_ nanofibers (II) and SnO_2_ without GO (I) to ethanol gas compared to other gases [182].

In et al. reported the fabrication of GO-SnO_2_ nanofibers for the detection of ethanol gas [182]. The process of preparing GO-SnO_2_ nanofibers involved electrospinning SnO_2_ nanofibers, followed by GO dip-coating and thermal annealing. The optimal operating temperature of the sensors was determined to be 300 °C, with a response of 85.3 (*R_a_*/*R_g_*) to 100 ppm of ethanol vapor (Table 4). Additionally, the functionality of the sensors persisted under high relative humidity conditions of 96%, showing a response of 51.75 to 100 ppm ethanol gas (Figure 20a). This impact from humidity decreases the response of the sensor by affecting conductivity, as water molecules compete with the target gas for adsorption sites [183]. The selectivity of the sensor for ethanol vapor was also demonstrated by testing against other gases such as ammonia, acetone, methanol, and ammonia acetate (Figure 20b). The interaction between graphene and SnO_2_ was proposed to contribute to the gas sensing mechanism, as electron transfer from SnO_2_ to graphene increases the number of active sites that are available for ethanol molecule adsorption [184,185].

### 5.4. Formaldehyde

**Figure 21:**
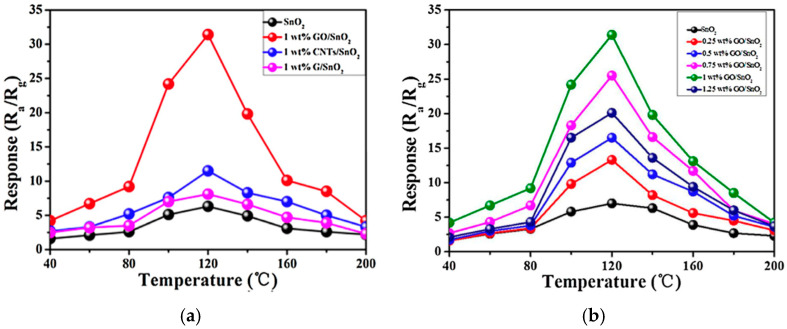
(**a**) Response of RGO-SnO_2_ sensor compared to RGO with other nanocarbons to 100 ppm of formaldehyde as a function of temperature; (**b**) Response of RGO-SnO_2_ sensor containing different RGO concentrations as a function of temperature [186].

Yang et al. investigated electrospun hollow SnO_2_ nanofibers with carbon materials, including graphene, carbon nanotubes, and graphene oxide, and the resulting nanofibers were examined as gas sensors for formaldehyde [186]. The study found that the GO-SnO_2_ sensors demonstrated superior gas response and selectivity to formaldehyde vapor, compared to SnO_2_ nanofiber sensors and SnO_2_ with other nanocarbons (Figure 21a). The optimal operating temperature was 120 °C, where the sensors containing 1 wt% GO achieved a response value of 32 (*R_a_*/*R_g_*) to 100 ppm of formaldehyde (Table 4), which is four times higher than that of nanofibers without GO (Figure 21b). The sensing mechanism of GO-SnO_2_ involves the interaction of gas molecules with the surface of metal oxides, leading to changes in electrical conductivity [187]. Hollow SnO_2_ nanofibers with porous morphology allow gas molecules to permeate, facilitating gas adsorption and electron transfer. With the addition of GO, the selectivity and sensitivity of the sensor are enhanced by lowering the energy barrier for electron transfer and providing active sites for oxygen species generation [188].

**Table 4:** Summary of graphene-based fiber gas sensors for VOCs.

Gas	Conc.	Material	Response	Temp.	Ref.
Acetone (C_3_H_6_O)	100 ppm	GO-WO_3_	35.9	375 °C	[173]
	200 ppm	RGO-ZnO	4	200 °C	[176]
	100 ppm	RGO/α-Fe_2_O_3_	8.9	375 °C	[178]
	500 ppm	RGO-PVDF	0.25	RT	[179]
Chlorobenzene (C_6_H_5_Cl)	4.72 μg/s	RGO-Cu	12.5 × 10^−6^ G	20 °C	[180]
Ethanol (C_2_H_6_O)	100 ppm	GO-SnO_2_	85.3	300 °C	[182]
Formaldehyde (CH_2_O)	100 ppm	GO-SnO_2_	32	120 °C	[186]

## 6. Graphene-Based Fiber Sensors for Detection of Multiple Gases

Thus far in this review, the focus has been placed on gas sensors specifically designed for the detection of individual gases. However, gas sensing systems capable of detecting two or more gasses are also extremely advantageous in real-world, complex environments. Similarly to single gas detection systems, sensing systems for multiple gases can be useful for different scenarios where it is desirable to detect many gases, such as environmental monitoring for air pollutants [189]. Furthermore, multi-gas sensors have been increasingly used in human health diagnoses based on breath, which can contain many trace gases, several of which are biomarkers for disease and adverse health conditions [190,191,192].

**Figure 22:**
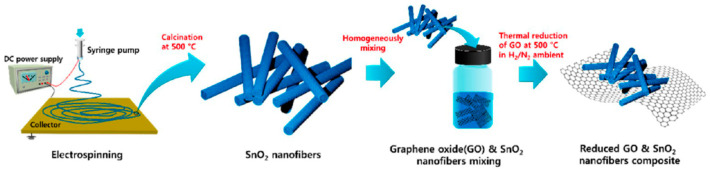
Schematic illustration of the preparation of RGO-SnO_2_ nanofiber composite [193].

Kim et al. reported a gas-sensing system capable of detecting H_2_S and acetone with RGO-SnO_2_ nanofibers [193]. SnO_2_ nanofibers were obtained via electrospinning and subsequently calcined via thermal treatment. The resulting nanofibers were combined with GO, followed by a thermal reduction to achieve an RGO-SnO_2_ nanofiber composite (Figure 22). Sensors containing 0.01 wt% of RGO exhibited a response of 34 (*R_air_*/*R_gas_*) to 5 ppm of H_2_S at 200 °C, while sensors containing 5 wt% RGO displayed a response of 10 to 5 ppm of acetone at 350 °C (Table 5). It was found that at low RGO loading concentrations, the sensing properties were predominately influenced by the SnO_2_ component, whereas the RGO component determined the electrical transport at higher RGO concentrations. Notably, the sensors were examined in a humid atmosphere at the respective optimal operating temperatures to investigate the interference of water vapor. As the sensors were able to detect the gases in humid air, this shows the potential of these sensors for breath analyzers that use acetone and H_2_S as biomarkers for the diagnosis of diabetes and halitosis [194,195]. This gas sensing system is notable as the loading of RGO in the nanofiber composite results in greater sensitivity to one gas, rather than the other. In addition, it underscores the challenges of gas sensing in the presence of interfering substances.

Fabricating gas sensors often encounter problems related to susceptibility to environmental changes, such as humidity and temperature [196]. A primary limitation of gas sensors is achieving high selectivity to detect a specific gas amid interfering substances, as cross-sensitivity remains a persistent challenge in both single-gas and multi-gas sensors, affecting accuracy in real-world conditions [197]. Addressing this problem involves controlling the morphology of sensing materials to enhance the detection of particular gases. It is necessary to optimize the sensing material by fine-tuning its surface properties and nanostructure to achieve better interaction with gases while taking into account the chemical composition, molecular size, and reactivity of the target gas molecule. It is significant to note that ongoing research involving gas sensors, including electronic nose technology, is exploring artificial intelligence and machine learning algorithms in sensing systems to enhance the accuracy and precision of gas detection and differentiation [15,198]. Further research is required to mitigate cross-sensitivity issues and facilitate precise discrimination between gases in multi-gas sensing applications.

Ren et al. also fabricated electrospun RGO-SnO_2_ nanofibers and studied their sensing behavior to NO_2_ and sulfur dioxide (SO_2_) under different intensities of UV light illumination [199]. RGO-SnO_2_ sensors were prepared through electrospinning, following calcination, and ultrasonic treatments, where different concentrations of SnO_2_ were combined with RGO, and the resulting sensors were investigated from dark to UV light irradiation with different light intensities. The sensor containing a mass ratio of RGO:SnO_2_ at 1:40 showed relatively similar responses of 23% and 22% ((*R_g_* − *R_a_*)/*R_a_* × 100%, where *R_g_* and *R_a_* are the resistance values in the gas and air, respectively) to 3 ppm of NO_2_ and 30 ppm SO_2_, respectively, in a dark environment at room temperature (Table 5). Interestingly, under 97 mW/cm^2^ of UV illumination, the sensor exhibited the highest response of 102% to NO_2_ but also the lowest response of 11% to SO_2_. In the presence of UV light, SnO_2_ absorbs UV light and collects photo-electrons, whereas RGO accepts these photo-electrons and facilitates charge transport. The enhanced selectivity is likely due to photocatalytic oxidation and photochemical desorption effects, leading to varied responses depending on the gas [200]. The findings of this study suggest that sensor selectivity can be improved by optimizing the intensity of excitation light, presenting the role of UV light in improving gas detection for specific gases.

**Figure 23:**
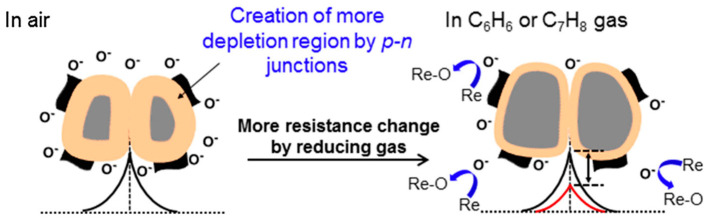
Schematic illustration of gas sensing mechanism of RGO/(Pt or Pd)/SnO_2_ (the change in the potential barrier is presented by the black and red curves) [201].

Kim et al. presented nanofibers composed of RGO and SnO_2_, loaded with platinum (Pt) or palladium (Pd), for the selective detection of benzene and toluene, respectively [201]. Unlike the previously discussed study that modulates graphene to target different gases, this approach optimized the gas-sensing properties of the sensor by varying the type of metal used. The sensors were fabricated by incorporating either Pt or Pd nanoparticles, grown via UV irradiation, into a SnO_2_ and RGO composite, which was then processed into nanofibers using electrospinning. The RGO/Pd/SnO_2_ sensors exhibited the highest sensitivity to benzene, with a response of 12.3 (*R* = *R_a_*/*R_g_*, where *R_a_* and *R_g_* are the resistances in the presence of air and target gas) at 5 ppm at 200 °C. In contrast, the RGO/Pt/SnO_2_ nanofibers demonstrated the strongest response to toluene, achieving a sensitivity of 16.0 at 5 ppm, which is 255% greater than their response to benzene (Table 5). This behavior can be attributed to toluene generating more hydrogen molecules than benzene, enabling Pt to dissociate toluene more efficiently, resulting in a stronger response in Pt-loaded sensors [202,203]. In the case of Pd-loaded sensors, Pd nanoparticles demonstrate higher catalytic activity for benzene decomposition owing to their lower adsorption energy [204,205]. Conversely, the adsorption of toluene by Pt is electronically favorable, while benzene dehydrogenation is thermodynamically unfavorable [205]. RGO also contributes to the gas sensing mechanism by absorbing electrons from adjacent SnO_2_, which increases the resistivity within the nanofibers, thereby reducing their conductivity and intensifying resistance modulation [124]. Electron flow through the connected SnO_2_ nanograins and p-n junctions at the SnO_2_ and RGO interfaces alters resistance as the depletion region contracts upon gas exposure (Figure 23).

**Figure 24:**
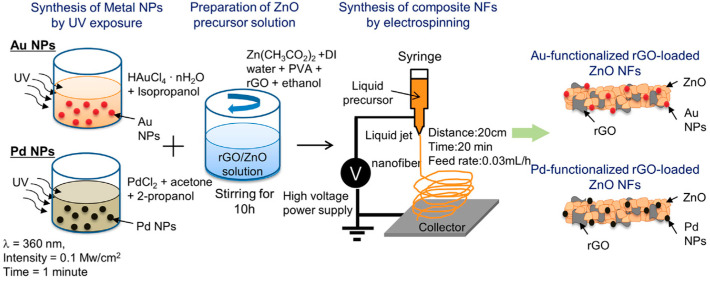
Schematic illustration of the synthesis of RGO/(Au or Pd)/ZnO [206].

The same research group also reported RGO and ZnO nanofiber sensors following similar methodologies for the gas sensing of CO and benzene [206]. The preparation of the sensors involved the synthesis of Au and Pd nanoparticles through UV radiation and incorporation with RGO and ZnO (Figure 24). The resulting composite solution formed nanofibers via electrospinning, and the as-spun nanofibers were subjected to a calcination treatment. The nanofibers containing Au exhibited a higher response to CO, whereas the sensors with Pd demonstrated a greater sensitivity to benzene. The RGO/Au/ZnO sensor showed a response of 23.5 to 1 ppm of CO, while RGO/Pd/ZnO had a response of 11.8 to 1 ppm of benzene at 400 °C (Table 5). This sensitivity is due in part to the high catalytic efficiency of Au nanoparticles for CO oxidation by lowering the oxidation barrier and the small kinetic diameter of CO, which allows molecules to permeate into the sensor and result in an amplified response. This gas sensing system is notable for its ability to achieve a stronger response to one gas over another by adjusting the type of metal nanoparticle employed.

Ruiz-Valdepeñas et al. also realized the sensing properties of graphene-doped SnO_2_ nanofibers, utilizing graphene synthesized through a liquid phase exfoliation process, whereby direct exfoliation of graphite was achieved in water-based solutions without the use of stabilizing agents [207,208]. The sensors consisted of electrospun nanofibers with a diameter of around 50 nm and nanoribbons approximately 1 µm in diameter deposited onto interdigitated electrodes. When exposed to various gases, the sensor exhibited responses exceeding 35% ((*R_a_* − *R*) × 100/*R*, where *R_a_* and *R* are sensor resistance under exposure to air and selected gas, respectively) for acetone and ethanol gases at temperatures ranging from 25–300 °C, with peak responses of approximately 85% to 4 ppm of acetone and 90% to 2 ppm of ethanol at 300 °C (Table 5). The sensor demonstrated negligible responses for CO and NO gases, thereby displaying a preference for acetone and ethanol. This behavior does not indicate selectivity for specific gases but rather shows a stronger sensitivity to certain gases compared to others. Although sensitivity is optimal at higher temperatures, varying the temperature allows for different sensor responses, facilitating their use in multi-sensor systems. It was proposed that the presence of graphene increases both the detection of the sensors at low temperatures and the response to gases. This enhancement was attributed to the existence of n-p heterojunctions that form potential barriers influenced by gas adsorption, while the porous structure of the nanofibers and nanoribbons improves gas penetration, thereby increasing sensitivity [209].

Cheng et al. investigated the effects of varying types of RGO on gas sensitivity performance by fabricating nanofibers from polyaniline, camphorsulfonic acid (HCSA), polyethylene oxide (PEO), and different RGO forms, including thermally reduced (trGO), chemically reduced (crGO), chemically reduced for 6 h (crGO-6), and chemically reduced for 24 h (crGO-24) [210]. These electrospun nanofibers were deposited onto interdigitated microelectrodes to fabricate sensors, which were then tested for responses to aliphatic alcohol vapors: methanol, ethanol, and 1-propanol. The sensor with crGO-6 exhibited the highest resistance modulation, showing the strongest response to 1-propanol, followed by methanol and ethanol. It exhibited responses of 22.6, 7.9, and 2.1 (Δ*R*/*R*_0_, where *R*_0_ is baseline resistance and Δ*R* is change in resistance upon exposure to analyte vapor) to 200 ppm of 1-propanol, methanol, and ethanol, respectively, at room temperature (Table 5), outperforming the other sensors containing differing RGO variants. Upon adsorption of vapor molecules, the nanofiber swells, increasing the separation between PANI chains, widening the electron transport gap, and increasing the resistance, with larger analytes amplifying this effect. The enhanced response of crGO-6 compared to crGO-24 suggests that hydrogen bonding between vapor molecules and RGO plays a role in resistance modulation and contributes to the overall sensing mechanism [211]. This study not only examines the impact of various reduction methods but also points to the effects of gas molecule size on the gas-sensing response.

**Figure 25:**
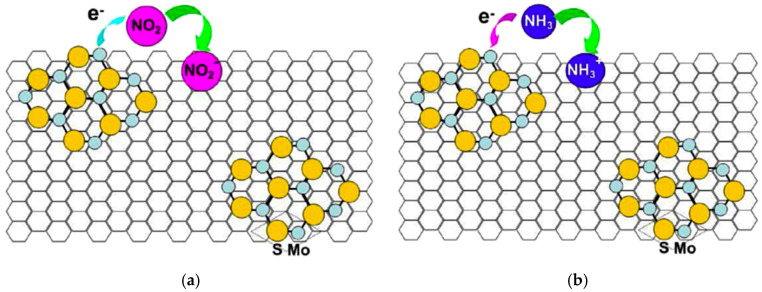
(**a**) Schematic illustrating the gas sensing mechanism of RGO-MoS_2_ composite fiber to (**a**) NO_2_ and (**b**) NH_3_ gas [212].

He et al. developed an RGO-MoS_2_ composite fiber with NO_2_ and NH_3_ gas-sensing properties [212]. The synthetic approach involved wet-spinning a composite containing GO and sodium molybdate, followed by treatment with L-cysteine, hydrothermal process, then thermal annealing. The resulting RGO-MoS_2_ fiber consists of MoS_2_ domains anchored onto the surface of graphene. It is observed that the sensor displayed a sensitivity of −85% (*S* (%) = 100 × Δ*R*/*R*_0_ = 100 × *R_g_* − *R*_0_/*R*_0_, where *R_g_* is the resistance under target gas exposure and *R*_0_ is the initial resistance under N_2_ exposure) to 100 ppm of NO_2_ and 100% to 100 ppm of NH_3_ gas (Table 5). RGO-MoS_2_ conjugates facilitate rapid charge transfer, leading to fast resistance fluctuations. When the sensor is exposed to NO_2_ gas, the p-type dopant accepts electrons from MoS_2_, resulting in a decrease in the resistance of the sensor (Figure 25a). When exposed to NH_3_ gas, an n-type dopant, electrons are donated to MoS_2_, resulting in an increase in the resistance (Figure 25b). This study demonstrates a gas-sensing system achieved by a singular fiber material that exhibits an inverse response to either NO_2_ or NH_3_. It should be noted that while selectivity between these two gases is high, the detection of either of these gases from the ambient atmosphere, where cross-selectivity poses challenges, was not tested in this study. However, these findings, particularly the sensitivity measured by resistance changes, warrant further study to identify potential response patterns that could enable differentiation between gases from the sensor. As demonstrated in this study, different gases interact uniquely with the sensing material, resulting in characteristic resistance changes. This distinction can be employed in sensors to improve pattern recognition and enhance the selectivity of sensing systems [213,214].

**Figure 26:**
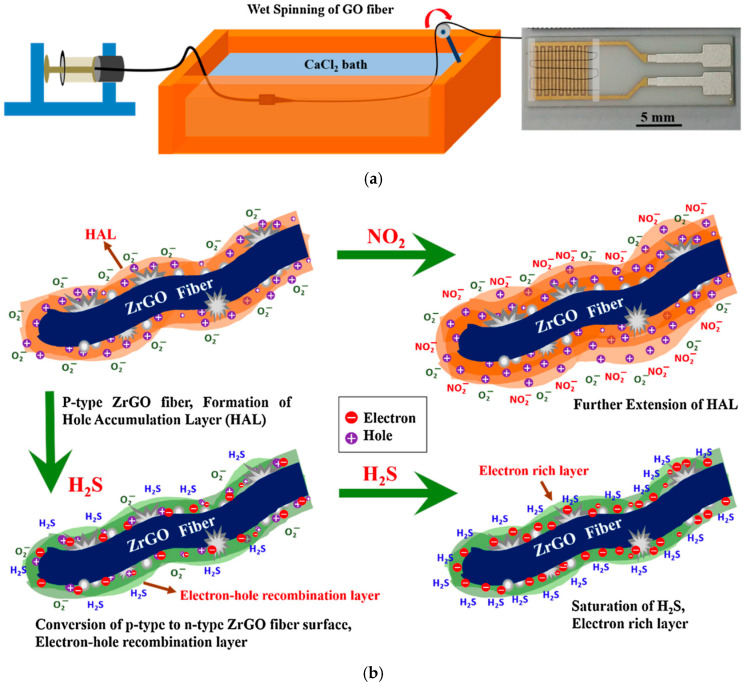
(**a**) Schematic illustration of wet-spinning of GO fiber and fiber sensor; (**b**) Schematic illustrating the sensing mechanism of RGO-ZnO fiber [215].

Yoo et al. reported fiber sensors composed of RGO and ZnO composites with the ability to sense NO_2_ and H_2_S gas [215]. The preparation of the fibers involved the wet-spinning of GO suspension into a coagulation bath containing calcium chloride, followed by hydrothermal treatment with zinc nitrate to obtain RGO-ZnO fibers (Figure 26a). The sensors were constructed by depositing the fibers onto interdigitated gold electrodes and adhering them with tape. The fiber sensor exhibited a sensitivity of 1.65 ((*R_a_* − *R_g_*)/*R_g_*] × 100 (%), where *R_a_* and *R_g_* are the resistances of the sensor material when air and gas were injected into the sensor, respectively) when exposed to 4 ppm of NO_2_ and 2.68 to 20 ppm of H_2_S at room temperature, respectively (Table 5). When exposed to CO_2_ and H_2_ gases, the sensor showed low sensitivity, thus demonstrating greater responses towards NO_2_ and H_2_S. The stability of the sensor was evaluated by subjecting the sensor to continuous NO_2_ and H_2_S exposure for 10 days, where the sensor performance was observed to be constant for both gases. The fiber sensor possesses p-type semiconductor characteristics, which causes the formation of a hole accumulation layer in the open air (Figure 26b). Electron transfer from the fiber increases hole concentration upon exposure to NO_2_. In contrast, exposure to H_2_S causes electrons to be transferred to the fiber and reduces the hole concentration, thereby inducing n-type behavior in the fiber [216,217]. The sensing mechanism is further influenced by the morphology of ZnO and RGO, which possess a high surface area of catalytic sites for gas adsorption and desorption. This work presents a promising platform for real-time human health monitoring, leveraging H_2_S as a biomarker, as well as in environmental monitoring, as NO_2_ is considered a hazardous gas. Crucially, the stability of these nanocomposites is demonstrated, marking a significant advancement towards the development of lightweight and robust sensors with potential applications in wearable electronics and automated portable devices.

Although graphene itself is known for its chemical stability and mechanical durability, the longevity of graphene-based fibers is influenced by interfacial properties between graphene and fiber-forming composite materials [218,219]. Ensuring long-term operational stability under various conditions is crucial for sensor performance reliability. While graphene itself demonstrates mechanical and chemical stability, the long-term stability of graphene-based composite fibers often depends on the non-graphene components, such as metal oxides or polymers [113,218]. In the case of polymer-containing composites, the durability of these fibers is influenced by the interaction between graphene sheets and the polymer matrix, which serves as the fiber-forming framework [218]. Therefore, the strain on this matrix plays a critical role in determining the overall stability and performance of the fiber. However, in composites consisting of metal oxides, graphene can enhance the structural integrity and improve the stability of the overall network owing to synergistic effects [220]. Continued research into these composite materials will aid in developing graphene fibers with optimal stability to achieve the best performance.

It is evident that sensor systems for multiple gases demonstrate significant potential across various applications, owing to their ability to target a wide range of gases. As mentioned, this versatility enables their use in diverse fields such as environmental monitoring, industrial safety, and medical diagnostics, where the detection of multiple gases simultaneously is crucial for accurate assessments and timely responses. The examples presented in this review showcase the current framework and obstacles to overcome for the expansion of more sophisticated systems with high sensitivity and selectivity.

**Table 5:** Summary of gas sensing systems for multiple gases.

Material	Gas	Conc.	Response	Temp.	Ref.
RGO-SnO_2_	H_2_S	5 ppm	34	200 °C	[193]
	Acetone		10	350 °C	
RGO-SnO_2_|UV light	NO_2_	3 ppm	102%	RT	[199]
	SO_2_	20 ppm	11%		
RGO/Pd/SnO_2_	Benzene	5 ppm	12.3	200 °C	[201]
RGO/Pt/SnO_2_	Toluene		16.0		
RGO/Au/ZnO	CO	5 ppm	35.8	400 °C	[206]
RGO/Pd/ZnO	Benzene		22.8		
Graphene-SnO_2_	Acetone	4 ppm	85%	300 °C	[207]
	Ethanol	2 ppm	90%		
RGO/PANI/HCSA/PEO	1-propanol	200 ppm	22.6	RT	[210]
	Methanol		7.9		
	Ethanol		2.1		
RGO-MoS_2_	NO_2_	100 ppm	−85%	RT	[212]
	NH_3_		100%		
RGO-ZnO	NO_2_	8 ppm	1.86	RT	[215]
	H_2_S		0.87		

## 7. Conclusions and Perspectives

This review highlights a decade of advancements in graphene-based composite fibers for gas sensing applications. It explores their preparation, fabrication into sensors, and gas sensing mechanisms, emphasizing graphene’s ability to enhance sensitivity and selectivity through its high surface area, electrical conductivity, and chemical tunability. These fibers also offer flexibility and mechanical strength, enabling integration into wearable and flexible electronics. The performance of these fibers in detecting various gases, including diatomic (H_2_, CO), triatomic (CO_2_, NO_2_, H_2_S), polyatomic (NH_3_), and VOCs (acetone, ethanol, formaldehyde) is detailed, with multi-gas sensing systems summarized for broader applications. As graphene-based fiber sensors continue to evolve, their seamless incorporation into everyday objects as wearable and portable devices holds great potential to enable real-time monitoring in fields such as industrial safety, environmental monitoring, and medical diagnostics.

Despite their promise, challenges persist. Environmental factors like temperature and humidity, as well as cross-sensitivity, impact real-world accuracy. Optimization of material morphology and surface properties is critical to enhancing selectivity and performance. Key hurdles in practical deployment include ensuring long-term stability, scalability for industrial production, and sustainable manufacturing. Stability depends on the interactions between graphene and other components within the composite, such as polymers or metal oxides. Scaling up production requires refining techniques like wet-spinning and electrospinning for consistent, high-quality output. Sustainability efforts must focus on renewable graphene production and recycling to minimize environmental impact.

Continued research is vital to overcoming challenges and driving innovation, paving the way for environmentally conscious, next-generation gas sensing technologies. This review has presented the versatility of graphene-based fibers and the significant potential these materials hold for gas sensing systems, highlighting the foundation established for the next breakthrough in the field.

## Data Availability

Not applicable.
